# The self-disproportionation of enantiomers (SDE): a menace or an opportunity?

**DOI:** 10.1039/c7sc05138g

**Published:** 2018-01-15

**Authors:** Jianlin Han, Osamu Kitagawa, Alicja Wzorek, Karel D. Klika, Vadim A. Soloshonok

**Affiliations:** a School of Chemistry and Chemical Engineering , State Key Laboratory of Coordination Chemistry , Jiangsu Key Laboratory of Advanced Organic Materials , Nanjing University , 210093 Nanjing , China . Email: hanjl@nju.edu.cn; b Department of Applied Chemistry , Shibaura Institute of Technology , 3-7-5 Toyosu, Kohto-ku , Tokyo 135-8548 , Japan; c Institute of Chemistry , Jan Kochanowski University in Kielce , Świętokrzyska 15G , 25-406 Kielce , Poland; d Department of Organic Chemistry I , Faculty of Chemistry , University of the Basque Country UPV/EHU , Paseo Manuel Lardizábal 3 , 20018 San Sebastián , Spain . Email: vadym.soloshonok@ehu.es; e Molecular Structure Analysis , German Cancer Research Center (DKFZ) , Im Neuenheimer Feld 280 , D-69009 Heidelberg , Germany . Email: k.klika@dkfz.de; f IKERBASQUE, Basque Foundation for Science , Alameda Urquijo 36-5, Plaza, Bizkaia , 48011 Bilbao , Spain

## Abstract

Herein we report on the well-documented, yet not widely known, phenomenon of the self-disproportionation of enantiomers (SDE): the spontaneous fractionation of scalemic material into enantioenriched and -depleted fractions when any physicochemical process is applied. The SDE has implications ranging from the origins of prebiotic homochirality to unconventional enantiopurification methods, though the risks of altering the enantiomeric excess (ee) unintentionally, regrettably, remain greatly unappreciated. While recrystallization is well known as an SDE process, occurrences of the SDE in other processes are much less recognized, *e.g.* sublimation and even distillation. But the most common process that many workers seem to be completely ignorant of is SDE *via* chromatography and reports have included all manner of structures, all types of interactions, and all forms of chromatography, including GC. The SDE can be either a blessing – as a means to obtain enantiopure samples from scalemates – or a curse, as unwitting alteration of the ee leads to errors in the reporting of results and/or misinterpretation of the system under study. Thus the ramifications of the SDE are relevant to any area involving chirality – natural products, asymmetric synthesis, *etc.* Moreover, there is grave concern regarding errors in the literature, in addition to the possible occurrence of valid results which may have been overlooked and thus remain unreported, as well as the potential for the SDE to alter the ee, particularly *via* chromatography, and the following concepts will be conveyed: (1) the SDE occurs under totally achiral conditions of (a) precipitation, (b) centrifugation, (c) evaporation, (d) distillation, (e) crystallization, (f) sublimation, and (g) achiral chromatography (*e.g.* column, flash, MPLC, HPLC, SEC, GC, *etc.*). (2) The SDE cannot be controlled simply by experimental accuracy and ignorance of the SDE unavoidably leads to mistakes in the recorded and reported stereochemical outcome of enantioselective transformations. (3) The magnitude of the SDE (the difference between the extremes of enantioenrichment and -depletion) can be controlled and used to: (a) minimize mistakes in the recorded experimental values and (b) to develop unconventional and preparatively superior methods for enantiopurification. (4) The magnitude of the SDE cannot be predicted but can be expected for compounds possessing SDE-phoric groups or which have a general tendency for strong hydrogen or halogen bonds or dipole–dipole or aromatic π–π interactions. (5) An SDE test and the rigorous reporting and description of applied physicochemical processes should become part of standard experimental practice to prevent the erroneous reporting of the stereochemical outcome of enantioselective catalytic reactions and the chirooptical properties of scalemates. New directions in the study of the SDE, including halogen bonding-based interactions and novel, unconventional enantiopurification methods such as pseudo-SDE (chiral selector-assisted SDE resolution of racemates), are also reported.

## Abstract 2

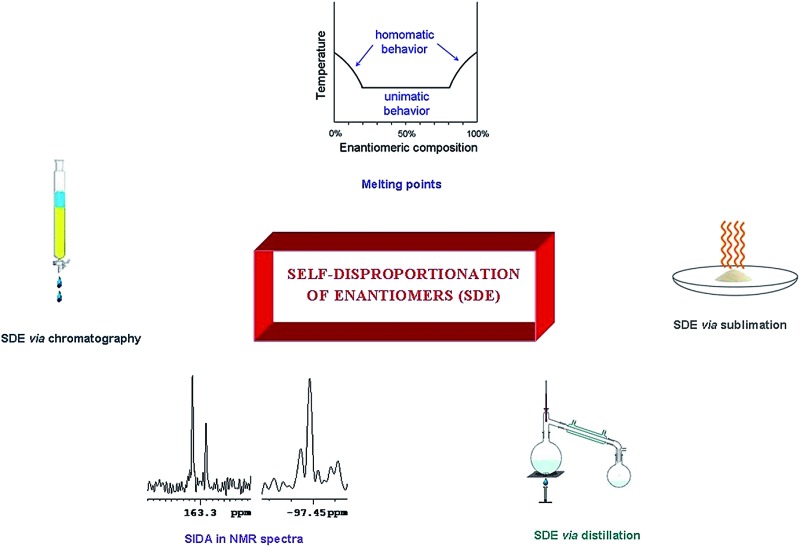
Herein we report on the well-documented, yet not widely known, phenomenon of the self-disproportionation of enantiomers (SDE): the spontaneous fractionation of scalemic material into enantioenriched and -depleted fractions when any physicochemical process is applied.

## 1 Introduction

The spontaneous fractionation of scalemic material into enantioenriched and -depleted fractions when a physicochemical process – any physicochemical process – is applied to a scalemate^1^ (a mixture of enantiomers that is neither 50 : 50 nor 100 : 0, *i.e.*, a sample that is neither racemic nor enantiopure) can result in the phenomenon of the self-disproportionation of enantiomers^2^ (SDE). Importantly, chirality is neither created nor lost during the process and if the collected fractions were recombined, the same enantiomeric excess (ee) for the recombined sample would be obtained as was present in the original sample prior to the process. Though the SDE phenomenon is well documented, it appears that it is generally not widely appreciated by researchers (at least for processes other than recrystallization) despite a large number of examples being described in several fine reviews on the SDE phenomenon involving processes other than recrystallization. These reviews include general accounts^3^ of the SDE phenomenon as well as one general review specific for fluorine-containing compounds,^4^ another dedicated to SDE *via* sublimation,^5^ as well as several dedicated solely to the SDE *via* achiral chromatography.^6^ Besides its scientific beauty and its profound importance for molecular chirality, the SDE has implications ranging from the origins of prebiotic homochirality to unconventional enantiopurification methods, though the risks of altering the ee unintentionally, regrettably, remain greatly unappreciated. In this report we examine the various physicochemical processes that have been reported to give rise to the SDE phenomenon as well as highlighting the pitfalls and potential advantages of the phenomenon.

While recrystallization is a well known occurrence of the SDE, other occurrences of the SDE are much less appreciated. Sublimation, if not widely appreciated, however is likely to be conceivable by most practitioners by analogy with recrystallization. Most practitioners, however, would probably be incredulous and highly skeptical that SDE could be possible by distillation, yet though extremely rare, there are confirmed reports of its occurrence. However, the process that is most common due to its all-pervasive use throughout chemical laboratories, but for which a majority of workers seem to be completely ignorant of, is SDE *via* chromatography. This can be either a blessing – as a means to obtain enantiopure samples from scalemates – or a curse as unwitting alteration of the ee leads to errors in the reporting of results, miscomprehension of the reaction pathway, hampering of the mechanistic interpretation, wrong evaluation of the practicality of (un)reported methodologies and other aspects of chiral-based studies. And in statistical terminology, both in the sense of Type I and Type II errors, *i.e.* methods which are purported to give good stereoselectivity but do not, as well as methodologies which are discarded (or used wrongly for interpretations) due to stereoselectivities which were evaluated as poor but which in fact are much better than realized. Thus the potential implications of the SDE phenomenon are of relevance to any area involving chirality – natural products, asymmetric synthesis, *etc.* Of note, reports of SDE *via* chromatography have covered all manner of structural types, all sorts of interactions, and all types of chromatography, *e.g.* gravity-driven column, flash, MPLC, HPLC, SEC, GC, *etc.* Furthermore, some functional groups provide a tendency for the molecules in which they are present to be much more prone to the phenomenon than others, and these groups have been given the moniker SDE-phoric groups.^7^ Groups identified as SDE-phoric include the amides of chiral amines,^7^–^14^ α-amino acid esters,6b,^9^,^11^ and β-amino acid esters^15^ as well as sulfoxides^16^–^18^ and compounds containing a trifluoromethyl group.^4^,^19^–^22^ Though advances have been made in predictability, challenges remain and this review updates the current situation. In addition, new directions in the study of SDE, including halogen bonding-based interactions and novel, unconventional enantiopurification methods such as pseudo-SDE (chiral selector-assisted SDE resolution of racemates), are also reported herein.

Since there is grave concern regarding errors in the literature, in addition to the possible occurrence of valid results which may have been overlooked and thus remain unreported, as well as the potential for the SDE phenomenon to alter the ee, particularly SDE *via* chromatography, we have been motivated to present this minireview as given the state of affairs, the SDE phenomenon could thus be construed as either an adverse (hence menace due to the ignorance of the SDE) or a favorable (hence opportunity if aware of the potential of SDE) occurrence. The major aim of this minireview is thus to provide readers with a compact and essential, yet comprehensive, overview of all aspects of the SDE phenomenon to make them equipped to handle any SDE-related problems as well as to potentially use the SDE phenomenon as a general, efficient enantiopurification method when the opportunity arises. Clearly though, workers should definitely heed the warnings that have been made regarding the potential menace that the SDE can pose.

## 2 Background and general aspects of the SDE phenomenon

The SDE term does not infer anything regarding the mechanistic aspects of the process, but rather refers only to the final outcome, *i.e.*, the concurrent formation of enantioenriched and -depleted fractions under totally achiral conditions external to the sample itself. Moreover, the precise mechanisms for the SDE phenomenon vary from one process to another, but the underlying precept, the preferential formation of homo- and heterochiral associates and the differences in their physicochemical properties, is common to all. Processes that have exhibited the SDE are outlined below in Section 3 Occurrences of the SDE phenomenon where selected examples are presented that highlight the problems that the SDE may cause and thus constitute a menace for workers, though the SDE can equally as well provide opportunities for workers to take advantage of in terms of novel, unconventional enantiopurification methods.

**Scheme 1:**
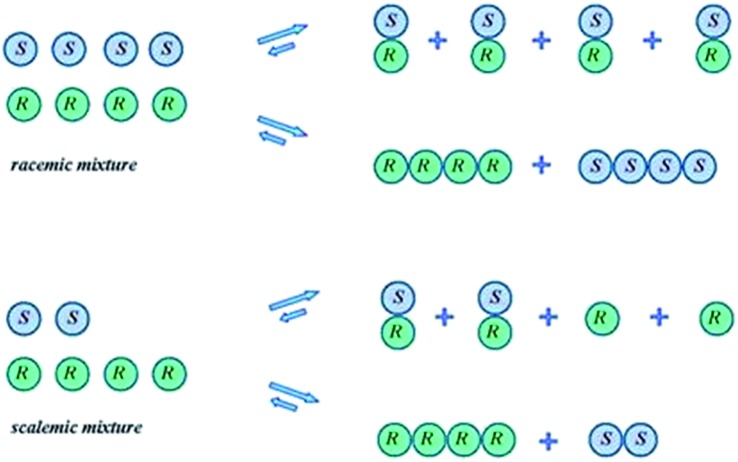
Possible equilibria for racemic and scalemic mixtures between monomers and homo- and heterochiral higher-order associates.

While SDE *via* crystallization is a well understood process and the reader is referred to the excellent literature on the topic, *e.g.*ref. 23, for solution only processes, *e.g.* SDE *via* chromatography, the understanding is less thorough. In solution, in contrast to the solid state, homo- and heterochiral higher-order associates are in a constant dynamic equilibrium with their single molecules and there is a persistent flux of molecules between the various states (Scheme 1). In the case of a racemic mixture, differences between the enantiomers will not occur irrespective of the position of the equilibria or the preference between homo- and heterochiral higher-order associates. In the case of a scalemic mixture, differences between the enantiomers will occur irrespective of the energies of the higher-order associates due simply to the differing concentrations. For most cases, there will be significant populations of monomers and both homo- and heterochiral higher-order associates. For dimeric associates, populations of homo- and heterochiral associates will be distributed according to K's while for oligomeric associates, the average size of the homo- and heterochiral associates as well as the populations can differ in the case of scalemic mixtures.

While it is instructive to consider the sample as being composed of a “racemic” and an “excess enantiomer” portion, the two portions are not temporally distinct in the sense that molecules belong to one or the other as they might be in a collection of crystals. Moreover, the position of such equilibrium and the relative population of higher-order species in solution is a function of a particular compound's structure and the energy of the corresponding intermolecular interactions as well as its concentration, which can change locally across the breadth of an eluting peak over the course of a chromatographic run say. Furthermore, the interactions of a given compound with the solvent can compete with, or interrupt altogether, the intermolecular forces leading to the formation of the homo- and heterochiral associations. Therefore, while the SDE phenomenon, in principle, always occurs, the observed magnitude can vary dramatically depending on a compound's structure and the prevailing conditions, whether it be chromatographic or otherwise. The magnitude of the SDE phenomenon can be expressed in a number of ways, one of which is to calculate the difference between the ee of the fraction with the highest obtained ee and the ee of the fraction with the lowest obtained ee (often the ee's of the first and the final eluting fractions):^8^

1 SDE magnitude (Δee) = ee_fraction with the highest ee_ – ee_fraction with the lowest ee_

Another parameter to indicate the strength of the phenomenon is the range of ee's over which the SDE appreciably occurs (with respect to a nominated minimum level of enantiopurity of the fraction(s) containing the enantiopure material and/or SDE yield (*vide infra*) and/or Δee):

2 SDE range (*R*_ee_) = ee_sample with highest ee exhibiting SDE_ – ee_sample with lowest exhibiting SDE_

The amount of enantiopure material that can be yielded by an SDE process is dependent on the ee of the starting material and in theory the maximum amount of enantiopure material obtainable in the case of SDE *via* chromatography is effectively very close to the amount of excess enantiomer present, *i.e.*, the ee:

3 Maximum theoretical yield for SDE *via* chromatography (*Y*_max,SvC_) ≈ ee

Thus the practical SDE yield as a percentage can be expressed as the isolated amount of the enantiopure material (with respect to a nominated minimum enantiopurity) divided by *Y*_max_ (converted to mass) and multiplied by 100:

4 SDE yield (*Y*_SDE_) = amount of the enantiopure material/(*Y*_max_ as mass) × 100

A number of studies have provided a theoretical basis for the SDE *via* chromatography.^24^–^27^ Based on these theoretical modelings – and fully consistent, in the main, with many observed results – it is worth noting some points regarding the SDE *via* chromatography phenomenon. Firstly, the complete separation of the excess enantiomer portion and the racemic portion is not possible. By contrast, the first eluting portion can, at least in theory, be obtained free of the other portion, *i.e.* if the excess enantiomer portion elutes first, an enantiopure fraction can be obtained, and conversely a perfectly racemic sample can be obtained if the racemic portion elutes first. And there are many examples of where enantiopure samples have been obtained from a chromatographic elution. However, the second eluting portion, at least in theory, cannot be obtained completely free of the first since the elutions of the two portions converge at the tail of the eluting peak. In practice these theoretical limits are of limited consideration as the contamination of either the first or second eluting portions with its complement can be negligible. The important point to note is that essentially enantiopure samples have often been obtained (in most cases as the first eluting component, but also on occasion as the second eluting component) and thus the SDE *via* chromatography process represents a practical and useful means to obtain enantiopure samples and can be included in the repertoire of methods to accomplish such. Indeed, SDE *via* chromatography actually has a decided advantage over fractional crystallization since fractional crystallization can only be applied to readily crystalline compounds while SDE *via* chromatography is applicable to all solids and liquids, which constitute the vast majority of all organic compounds. Quite interestingly, a sizeable difference in the energies of the homo- and heterochiral molecular associations is not required24a,d,^25^,^28^ for the SDE *via* chromatography phenomenon to occur in contrast to the other processes where the SDE phenomenon occurs.

Interestingly, it is less clear regarding the optimum ee of the sample for the maximum effect of the SDE *via* chromatography to be expressed, and like eutectic points (ep's) in fractional crystallization, it seems to vary considerably with the analyte and the particular conditions of the chromatography that are applied and thus there is not a universal optimum. As is apparent from the above discussion, either the excess enantiomer portion or the racemic portion of the sample can elute first, but it is worth emphasizing that this is not fixed for each particular compound and depending on the applied chromatographic conditions (*e.g.* solvent, stationary phase, *etc.*), the order of the portions can be reversed. At present, elution order is not universally predictable except in extreme cases, but it would be advantageous to be able to manipulate the system deliberately, *i.e.* to select which portion elutes first, as it can facilitate the means to obtain the desired portion (generally this will be the enantiopure material rather than racemic material) free of the second eluting component since the first eluting component more often than not can be obtained more free of the second eluting component. The latter point is also a general chromatographic outcome and not one specific to the SDE *via* chromatography phenomenon it is worth noting.

If the desire is to suppress the SDE *via* chromatography phenomenon, then disruption of the intermolecular interactions is nominally the approach to take, *e.g.* if the dominant intermolecular interaction of the analyte appears to be based on hydrogen bonding or dipole–dipole interactions, then the use of polar solvents, particularly those with a capability of forming intermolecular interactions based on hydrogen bonding or dipole–dipole interactions, is the obvious approach to take. Alternatively, if the desire is for expression of the phenomenon to take advantage of the possibility of effecting enantiopurification, then clearly polar solvents should be avoided or their inclusion minimized in such cases. One of the most effective practical means of increasing the prospect of the phenomenon occurring is to increase the concentration of the analyte to favor the formation of molecular associations. While reduction of the temperature should also favor occurrence of the phenomenon, it will generally be impractical to do so considering the usual conditions under which practitioners conduct chromatography and the likely limitations of effectively inducing a significant shift in the equilibrium at a practical level.

Finally, the interaction between enantiomers leading to the formation of homo- and heterochiral associates resulting in a perturbation of the ee is not restricted to purely physical processes. This aspect is explored Section 5.1 NLE's in asymmetric catalysis but of particular interest is the asymmetric autocatalysis in organocatalytic reactions where similar intermolecular interactions and also based on the formation of hydrogen bond-based complexes have been postulated^29^ to account for the observed results in an asymmetric Mannich reaction. Induction by the product was effected by competition between the homo- and heterochiral dimers of the product for the substrate to form a new complex consisting of the substrate and one product molecule, which was then the reactive species with the enol. Interestingly, by DFT calculations, the homo- and heterochiral dimers were of equal energy.

## 3 Occurrences of the SDE phenomenon

### 3.1 SDE *via* force field

Chiral crystalline compounds can adopt one of three basic arrangements of their constituent enantiomers within the crystallographic unit, whereby either equal numbers of the two enantiomers are present suitably arranged, just one of the two enantiomers is present, or anomalous amounts of the two enantiomers are present and randomly arranged.^30^ Compounds that preferentially adopt these basic arrangements under particular conditions are termed racemates (racemic compounds), conglomerates, and solid solutions, respectively.^23^ Which particular crystallographic structure is favored by an organic compound is unpredictable, but about 90–95% of chiral compounds crystallize as racemates while only an insignificant number of compounds crystallize as solid solutions.^23^ This overwhelming preference for racemates is thermodynamically driven as the two mirror-image enantiomers can usually form more close-fitting interactions^31^ and on average, racemic crystals are generally more stable and have higher melting points and densities in comparison with their corresponding enantiopure crystals.^32^ This generalization is known as Wallach's rule.^31^ Crystalline scalemates formed by complete deposition from a scalemic solution, if they are a racemic compound, are necessarily comprised of a mixture of racemic crystals and enantiopure crystals.

A quite remarkable demonstration of the possibility to separate a mixture of racemic crystals and enantiopure crystals due to their disparate densities was realized for a sample of crystalline scalemic (*S*)-alanine by density gradient ultracentrifugation.^33^ Using a 50.8% w/w Nycodenz solution, the separation of the racemic and enantiopure crystals was conducted using 100 mg of a 1 : 1 mixture of (*S*)- and (*R*/*S*)-alanine crystals allowing the separation and simple collection by filtration of enantiopure crystals after 2 h of centrifugation with an SDE yield of 50% (75–90% SDE yields were obtained from smaller scale runs and applying 21 h of centrifugation). The authors claim that SDE *via* centrifugation has great feasibility for large-scale practical separations as the procedure is operationally simple, cost efficient, and fully predictable and can be specifically tuned with respect to the difference in densities of the enantiopure and racemic crystals. Of note, this technique can be used on powders and does not require well-formed crystalline material nor for the material to be dissolved for the determination of ee, which is of particular importance in the case of chiral nanocrystals.

**Scheme 2:**
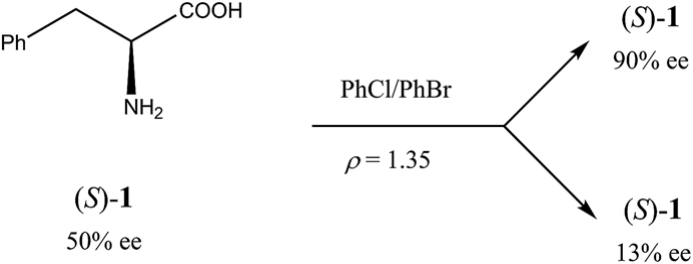
SDE *via* force field and application to (*S*)-phenylalanine (**1**) of 50% ee.

Another occurrence of the SDE *via* force field was described^34^ using gravity-driven dispersion within a fixed density medium for three compounds, *viz.* phenylethyl ammonium hydrogen fumarate, 2-amino-1,3-dihydroxy-1-(4-nitrophenyl)-propane, and phenylalanine (**1**). While for the first two compounds enantiodifferentiation was only modest (Δee 16–18% for fumarate and 39% for the diol), in the latter case it was particularly successful (Scheme 2). In this case, a finely powdered mixture of racemic and enantiopure crystals of **1** prepared by the complete evaporation of a scalemic solution of 50% ee was suspended in a mixture of chlorobenzene and bromobenzene with a specifically prepared density (*ρ*) of 1.35 g mL^–1^. After a couple of hours, the denser (*ρ* > 1.35 g mL^–1^) racemic crystals gravitated to the lower reaches of the fluid while the lighter (*ρ* < 1.35 g mL^–1^) enantiopure crystals floated towards the upper reaches. Samples of phenylalanine (**1**) could be isolated simply by separation of the fluid layers followed by filtration to yield material of 13% ee from the lower portion and 90% ee from the upper portion.

The ingenuity, cost-effectiveness, and outright simplicity of these processes should be highly motivating for the development of general and practical large-scale enantiomer purification procedures, especially on an industrial scale, considering that 90–95% of chiral compounds crystallize as racemates and that the SDE *via* force field is potentially a more efficient and cost-effective method than fractional crystallization. Thus it is surprising that only an extremely limited number of reports exist, and moreover, these reports seem to be almost completely unknown to the wider scientific community and they await due recognition and appreciation. The particular advantage that the SDE *via* force field holds over other permutations of the SDE is that it can provide, potentially, Δee's of 100%. In short, SDE *via* force field represents enormous untapped potential.

### 3.2 SDE *via* phase transitions

#### 3.2.1 Solid–liquid (crystallization and precipitation)

Another major manifestation of the differences in crystallographic structure between racemic and enantiopure crystals is their solubility. This property was noticed and used since practically the dawn of chemical science. Nowadays the physicochemical and thermodynamic rational of scalemate crystallization has received quite accurate description being routinely applied in the chemical industry and within laboratory settings. Whether or not homo- and heterochiral interactions are present in solution during a recrystallization is likely to be inconsequential as the solution-phase interactions are likely to be so much weaker than the crystallographic forces. Thus the preference between homo- and heterochiral associations that is determinant for the outcome of the process lies very much in the solid state.

Assuming that the readers are well-aware of the crystallization in its application for chemical and enantiomeric purifications, in this section, we will focus on some less commonly known misconceptions and shortcoming of this technique. First of all, in the minds of most chemists the “optical purification by recrystallization” is closely associated with only one part of the process – the preparation of the enantiopure compound, while the second part – the racemic or enantiodepleted product – is not usually considered. As discussed in the previous section, racemic crystals are generally more stable, denser, and less soluble than their enantiopure counterparts, therefore preparation of the racemic form from a scalemic sample is usually easier and a more feasible prospect. Thus, it is suggested to call the process “enantiopurification by SDE *via* crystallization”, the term which accounts for the resulting enantioenriched and -depleted fractions. Another issue one should remember is that about 90% of organic compounds are liquids or of low crystallinity rendering crystallization of rather limited area of application. In industry this limitation is dealt with by specifically making highly crystalline forms, regardless of the extra synthetic steps and additional cost. Furthermore, effectiveness of the enantiopurification by SDE *via* crystallization depends strongly on the starting ee and ep (the relative merits of the two approaches are compared further on in Section 4.2 New directions and novel, unconventional enantiopurification methods), being virtually inefficient generally for samples of less than 70% ee. Moreover, on average, the yields of recovered enantiomerically pure form are usually less than 50% and may take several recrystallizations to prepare samples of >99% ee. Finally, despite the fact that crystallization of scalemates is physicochemically well-understood, the choice of the appropriate solvent is a rather unpredictable endeavor with success not being guaranteed. Nevertheless, the enantiomeric purification by SDE *via* crystallization is widely used, not because of any inherent attractive features of the process, but because of the lack of other alternatives aside from the recrystallization of specially prepared diastereomeric derivatives or HPLC using chiral stationary phases. It is considered that developing an appreciation of the other cases of the SDE *via* phase transitions or achiral chromatography is highly desirable to overcome the current limitations in the selection of techniques for enantiopurification.

#### 3.2.2 Solid–gas (sublimation)

**Scheme 3:**
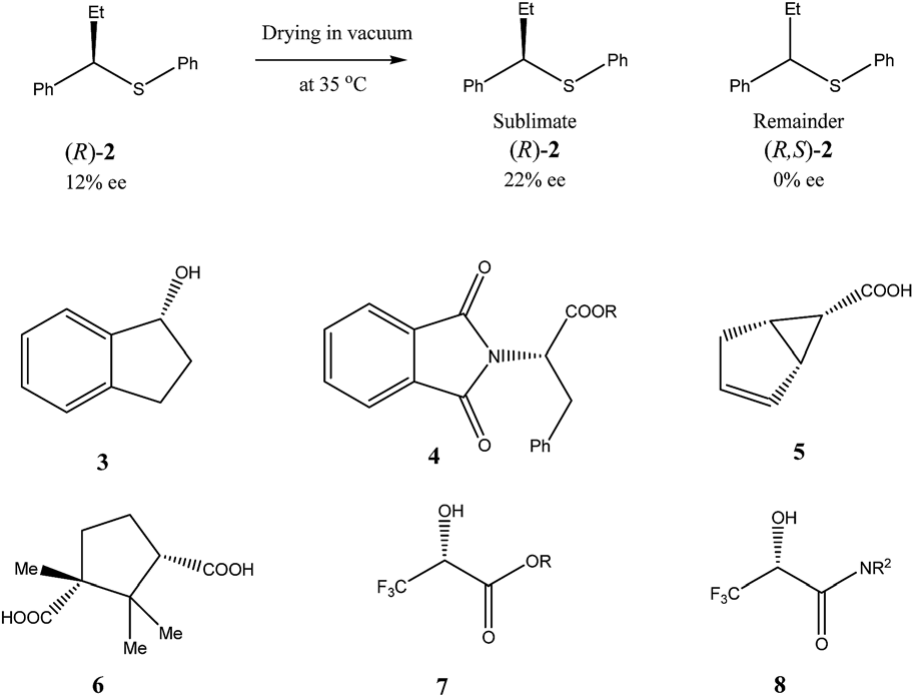
Examples of some compounds showing high magnitude of SDE *via* sublimation.

Similar to the SDE *via* force fields and crystallization, the SDE *via* sublimation stems from the differences in crystallographic structures between racemic and enantiopure crystals. Thus, besides different densities and solubilities, racemic and enantiopure crystals have different sublimation rates. However, this latter property was really virtually entirely overlooked and still remains one of the least studied areas of the SDE phenomenon. Thus, the earliest instances of the SDE *via* sublimation were stumbled upon purely accidentally when the observed stereochemical outcome of enantioselective reactions defied any logical explanation requiring detailed examination. For example, during the work on asymmetric synthesis of (*R*)-α-ethylbenzylphenyl sulfide (**2**) (Scheme 3)^35^ the authors noticed that optical purity of the samples of compound **2** obtained, depended on the time the samples were subjected to routine drying in vacuum. It was discovered that the faster subliming fractions of sulfide **2** had considerably higher optical purity as compared with the original sample, while the reminder was optically depleted. Completely racemic remainder, starting from the sample of 12% ee, was observed after 85 h of sublimation at 35 °C. A pedagogical aspect of this study is that unwary workers could possibly describe the stereochemical outcome of this catalytic enantioselective reaction as either racemic or highly enantioselective depending on whether sublimed or nonsublimed material was selected for analysis. Another important observation made in this work was the sublimation experiment conducted at 48 °C, the temperature at which the starting material was completely molten. It was found that both the sublimate and the remainder had the same enantiomeric composition as in the original sample. This experiment demonstrated importance of maintaining the crystalline state of a compound to realize the SDE *via* sublimation. Finally, the authors also conducted a series of experiments comparing sublimation and fractional crystallization to determine which procedure can provide for the most efficient separation of racemic form (*R*/*S*)-**2** from the excess enantiomer (*R*)-**2**. Their conclusion is: “It is also very clear that the sublimation method is by far the more efficient, taking consideration of the largest yield of the active material obtainable in the form of its greatest purity. From the point of view of simplicity of handling and obviating the necessity of finding the most appropriate solvent, as behooves the use of fractional crystallization procedures, sublimation is again the method of choice whenever it can be applied” and “the sublimation approach to separation of the active component may be attempted without untoward difficulties”.

**Scheme 4:**
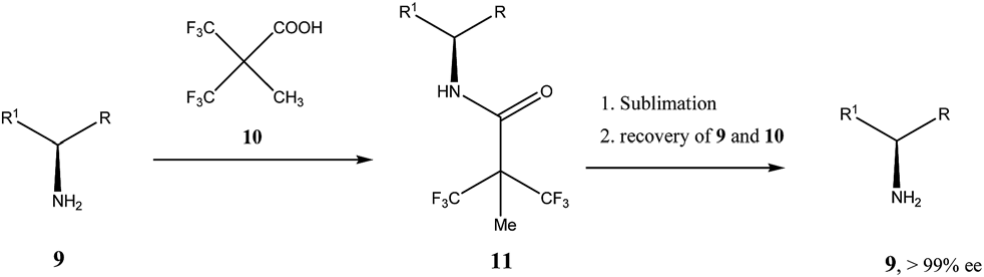
Enantiomeric purification of amino compounds using (hexafluoro)pivalic acid **10** as the sublimation enabling tag.

Very similar cases of an accidental discovery of SDE *via* sublimation were reported for compounds **3–6**^36^ when the crude products of enantioselective reactions were subjected to routine drying in vacuum or even during workup procedure using rotary-evaporation.^37^ Of particular interest are derivatives of trifluorolactic acid **7** and **8** which readily sublime at slightly elevated temperatures^38^ and can be used as model compounds to study various aspects of the SDE *via* sublimation.^39^ In this regard, one may agree that the usually low volatility of organic compounds would limit the general application of SDE *via* sublimation as an unconventional enantiomeric purification method. However, this impediment can be overcome by using a unique property of fluorine to influence physicochemical properties of organic compounds, such as melting points and sublimation.^21^,^40^ For example, (hexafluoro)pivalic acid **10** (Scheme 4) was proposed^41^ to be used as a sublimation enabling tag to modify physicochemical properties of various compounds, in particular those which are liquids or possess low volatility. In a representative case liquid amine **9** was transformed to amide **11** showing high crystallinity and exceptional volatility, thus allowing sublimation at ambient temperature and pressure. Amide **11**, of original 70.4% ee was simply spread over a Petri dish leaving enantiomerically pure remainder after 47.5 h. In this case the racemic portion of the original sample sublimed noticeable faster allowing for such unprecedented enantiomeric purification under ambient conditions.

Besides the discussed compounds **2–8** (Scheme 3) and specially designed derivatives **11**, SDE *via* sublimation was reported for naturally occurring mandelic acid,36*b* the highly popular commercial drug ibuprofen^42^ and some α-amino acids.^43^ It should be emphasized that since the SDE *via* sublimation originates from the differences in crystallographic structure between racemic and enantiopure crystals, it has to be expected for all compounds crystallizing in these solid forms. Thus, the relatively small number of examples in this area reported so far is because of (a) the usually low volatility of organic compounds and (b) a lack of knowledge of this phenomenon among chemistry practitioners. Due to the paucity of research data on the SDE *via* sublimation, the physicochemical description of the process is still quite poorly understood as the attempts to extrapolate “the eutectic composition”, in analogy to the melting eutectic, to sublimation were quite unsuccessful.^23^,^44^

One may conclude that the data reported thus far, show the potential of the SDE *via* sublimation as a viable alternative to conventional crystallization. However, it is still in its infancy as the amount of results available in the literature is very limited and somehow controversial as the commonly agreeable standards and procedures for reproducible sublimation experiments are yet to be established. Nevertheless, its operational simplicity, convenience and therefore potential economical practicality, in particular for large-scale separations, bode well for its wide spread application as an unconventional enantiomeric purification technique. In particular, with no need for a solvent and a predictable optimization, sublimation has quite attractive features for a greener and more economical approach.

#### 3.2.3 Liquid–gas (distillation)

**Fig. 1:**
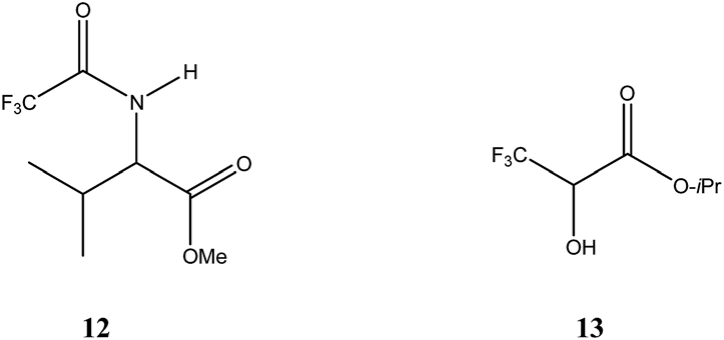
Structures of *N*-trifluoroacetyl valine methyl ester (**12**) and isopropyl (3,3,3-trifluoro)lactate (**13**).

A result that almost defies belief is SDE *via* distillation and early reports^45^ of such events were treated with due skepticism, not helped by the fact that early results were experimentally unconvincing, as were the unpersuasive theoretical arguments put forward. Consequently, these reports were generally largely dismissed.^46^ In the most authoritative book in the area of chirality it is clearly stated23*a* that “distillation of a partially resolved mixture is an operation that cannot lead to a modification of the enantiomeric purity”. While the basic principles of physical chemistry in the book are unquestionably correct, the generalized conclusion of improbability of the SDE *via* distillation only applies to compounds evaporating as monomers. The analysis therein did not consider cases of compounds capable of very strong intermolecular interactions and for these cases, simple classical thermodynamic considerations are insufficient. Indeed, two clear cases of SDE *via* distillation have been reported. The first unequivocal example was reported^47^ in 1989 for *N*-trifluoroacetyl valine methyl ester (**12**, Fig. 1), followed by a more meticulous account^48^ in 1996 for isopropyl (3,3,3-trifluoro)lactate (**13**). While a difference in bps was not discernible in the case of **12**, for **13**, the difference in bps between the racemate and the scalemate with the highest bp (a plateau region *ca.* 50–70% ee) is an astonishing 50 °C. Moreover, the distillate of **13** could either be enantioenriched or -depleted relative to the material in the distillation pot depending on its ee. For example, a sample of 74.1% ee yielded a distillate of 81.7% ee while a sample of 40.2% ee yielded a distillate of 33.2% ee. Thus, in complete analogy to the recrystallization of a racemic compound, an ep can be declared – at *ca.* 60% ee in this example – based on the bps and the consequent change in the ee of the distillate. Insufficient data was presented^47^ to assess whether an ep existed in the case of **12** but clearly its behavior is different to that of **13** with regard to the bps.

To account for the behavior of **13**, extensive intermolecular interactions must be present in at least the liquid phase, *viz.* hydrogen bonding, and their occurrence was evident by IR^48^ and also by low-angle X-ray diffraction^49^ studies. The strength of the hydrogen bonding and the extent of the hydrogen bonding network or repulsive properties between the molecules, in particular between CF_3_ groups^50^ are clearly accentuated by the trifluoromethyl group as the non-fluorinated analogue failed to exhibit SDE *via* distillation.^48^ But if there was simply a strong and overwhelmingly heavy bias towards heterochiral (homochiral) association, then it would be expected that at all times the distillate would be enriched in the excess enantiomer (racemate). Since this is not the case, either one or other of the associations is strongly preferred but with sufficient dependence on concentration, or the difference between the two types of association is not that dramatic, or the interactions are more complex than simple dimeric associations (*e.g.* long homochiral chains as evidenced in the solid state49b,^51^ and perhaps also in the liquid state49*a*), possibly also compounded by gas-phase intermolecular interactions which have been postulated but not proven.^48^ IR spectra of low (17%) and high (75%) ee samples of **13** seem to indicate that homochiral associations are indeed strongly favored.^48^ Certainly the very strong preference for homochiral interactions by **13** was not only shown in the solid state,49b,^51^ so much so that it is difficult to even obtain racemic crystals, but also by size-exclusion chromatography (SEC)^51^ (*vide infra* Section 3.3.5 SEC) and the compound has been considered^51^ an extreme case in this sense. But whether **13** is exclusively dominated by homochiral associations has yet to be determined. Nonetheless, since **13** has been shown to possess such a strong tendency for homochiral association, it might be considered that the liquid scalemate might be expected to behave as a conglomerate with the presumption of a high degree of homochiral association though one plausible rationalization of the distillation results might follow from a perspective of colligative properties as follows: at high ee above the ep, the minor enantiomer has its vapor pressure suppressed more than the excess enantiomer thus resulting in enantiomeric enrichment in the gaseous phase and hence the distillate has a higher ee than the starting sample. At low ee below the ep on the other hand, the vapor pressures of the two enantiomers are similarly depressed, *i.e.* with similar concentrations in the gaseous phase and enantiomeric depletion occurs in the gaseous phase and hence a more racemic distillate results.

Thus far, these observations of SDE *via* distillation have defied definitive explanation, and causes have been ascribed to both kinetics^47^ and thermodynamics^48^,49a though contributions from both are likely. Nonetheless, these results are truly astonishing and are surely most fascinating for practitioners, if not likely to be a method put into practice for enantiopurification. Nor are they a cause for concern in terms of unintentionally altering the ee of a sample during the course of a purification given that the likelihood of SDE *via* distillation is likely to be only a very rare occurrence, but they do demonstrate the extraordinary results that can occur by way of the SDE phenomenon.

### 3.3 SDE *via* achiral chromatography

#### 3.3.1 HPLC

**Fig. 2:**
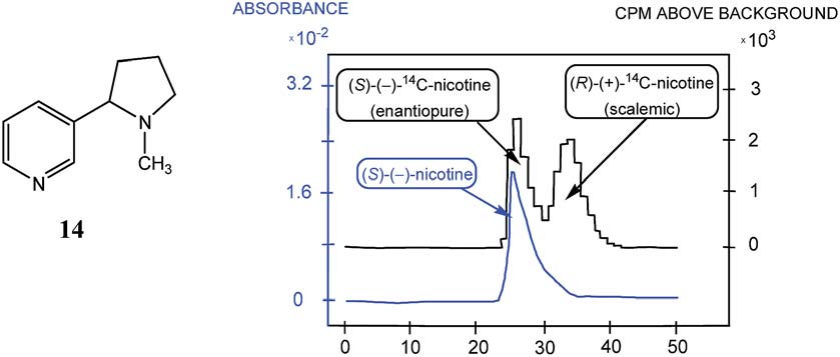
The HPLC traces (blue trace, UV detection; black trace, radiodetection of ^14^C by scintillation counting of fractions) obtained for the analysis of a mixture of radiolabeled (*rac*)-^14^C-2′-nicotine (**14**) with unlabeled (*S*)-nicotine (**14**). The first peak in the radiochromatogram is the selective detection of labeled enantiopure (*S*)-**14** while the second peak is the selective detection of a scalemic mixture of labeled **14**. The first clear peak in the UV-based HPLC trace is a mixture of labeled and unlabeled enantiopure (*S*)-**14** while the second indistinct peak overlapped with the first is a mixture of labeled and unlabeled “racemic” **14** (actually scalemic but tending to racemic and the ee of the unlabeled **14** arising from the enantiopure **14** and the labeled **14** is not the same as the ee of the labeled **14** originating from the labeled **14**). The following conditions were applied for the HPLC: column, Partisil PXS CSX; eluent, 0.12 M AcONa–MeOH (75 : 25); pH, 6.8; flow rate, 2.0 mL min^–1^.

The first example of SDE *via* HPLC was reported in 1983 by Cundy and Crooks^52^ where HPLC was performed using either reverse-phase Partisil PXS ODS or cation-exchange Partisil PXS CSX columns and the analyzed mixture consisted of radiolabeled (*rac*)-^14^C-2′-nicotine (**14**, Fig. 2) and enantiopure (or enantioenriched) unlabeled (*S*)-(–)-nicotine (**14**). The radiochromatogram resulting from the HPLC (Fig. 2) was considered rather incredible as it showed two distinct peaks instead of the anticipated single peak as expected for a chemically pure sample. The first peak in the radiochromatogram is a mixture of labeled and unlabeled enantiopure (*S*)-**14** while the second peak is a mixture of labeled and unlabeled “racemic” (actually scalemic but tending to racemic) **14**. The ee's of the radiolabeled **14** and the unlabeled **14** within the “racemic” peak are not equal and depend on the isotope incorporation level of the radiolabeled **14** sample, the proportions of labeled and unlabeled **14** that were mixed prior to chromatography, and the amount of (*S*)-**14** removed to the first eluting peak. Thus, after separation of the excess *S* enantiomer from the racemic portion, it will have reduced activity compared to half of the corresponding amount of the original radiolabeled **14** sample as some of radiolabeled *S* enantiomer will necessarily be in the racemic portion peak.

**Fig. 3:**
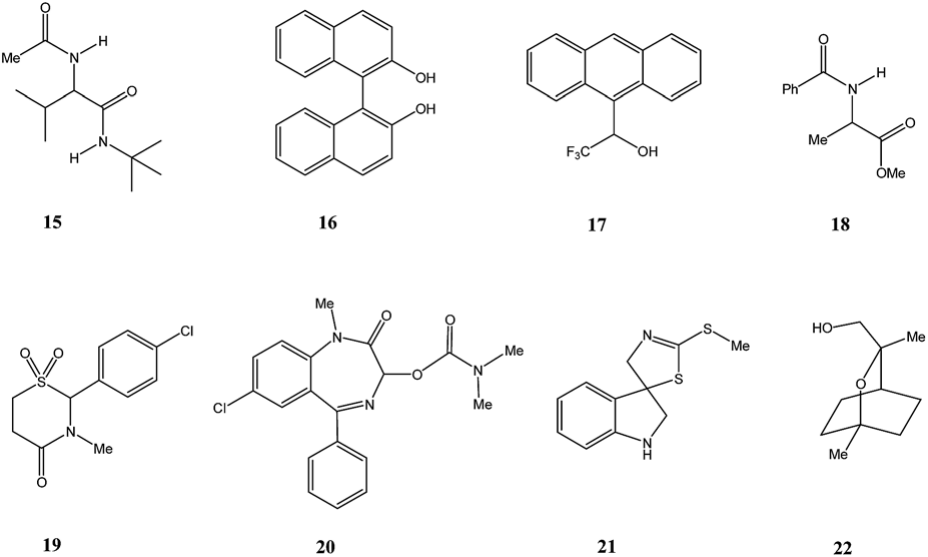
Structures of compounds **15–22** that have displayed SDE *via* HPLC.

This finding was so unexpected and astonishing that the authors repeated the procedure several times to confirm the discovery of a novel phenomenon, the separation of racemic and enantiopure forms of the same chemical compound under the conditions of achiral chromatography. Four years later, the Dobashi group reported^53^ very similar results for HPLC using achiral silica gel of a mixture of radiolabeled ^14^C-*N*-acetyl valine *tert*-butyl ester with unlabeled enantiopure *N*-acetyl valine *tert*-butyl ester (**15**, Fig. 3). The same pattern was observed for the separation of racemic and enantiopure portions in these experiments suggesting that the mere curiosity reported by Cundy and Crooks might have greater generalized significance. This work was followed by numerous reports supporting the notion that SDE *via* achiral HPLC can be readily observed for virtually any chiral compound. In particular, the separation of the excess enantiomer from the racemic portion of a scalemate has been achieved^54^ by HPLC using aminopropyl silica gel (LiChrosorb®-amine) as the stationary phase for scalemic samples of 1,1′-bi-2-naphthol (**16**), 1-anthryl-2,2,2-trifluoroethanol (**17**), and *N*-benzoyl alanine methyl ester (**18**) as well as the drugs chloromezanone (**19**) and benzodiazepine camazepam (**20**). Naturally occurring compounds were also observed to undergo SDE *via* HPLC, *e.g.* spirobrassinin (**21**)^55^,^56^ and 9-hydroxy cineole (**22**).^57^

The discovery by Cundy and Crooks of SDE *via* achiral HPLC opened a new direction in scientific research and initiated similar studies for scalemic samples under all other known types of achiral chromatography (discussed in the following sections). It is worth noting that Cundy and Crooks were also the first to point out the immense practical application of the SDE *via* achiral chromatography as a new enantiopurification approach, in particular in relation to the preparation of enantiopure samples of highly expensive ^14^C-radiolabeled enantiomers of nicotine, albeit at the cost of reduced activity. The occurrence of the SDE *via* HPLC for natural products, first reported^57^ in 1991 for the 1,8-cineole metabolite 9-hydroxy cineole (**22**), is, in addition to scientifically fascinating, also alarming given that HPLC is routinely used to isolate natural products. Thus, the application of HPLC has the potential to result in alteration of the enantiopurity of isolated material from a natural source, either rendering it more enantiopure or more racemic than it otherwise is in its natural state, and consequently leading to errors in the reported data and possibly in the interpretation of biochemical pathways.

#### 3.3.2 MPLC

Reports of SDE *via* achiral medium pressure liquid chromatography (MPLC) are infrequent in comparison to HPLC6*b* (see Section 3.3.1 HPLC), the major reason being that MPLC is primarily used for preparative separations and not as an analytical technique.^58^ Similarly to HPLC though, most reports of SDE *via* MPLC have been serendipitous observations during the course of purifying a scalemic sample.

**Fig. 4:**
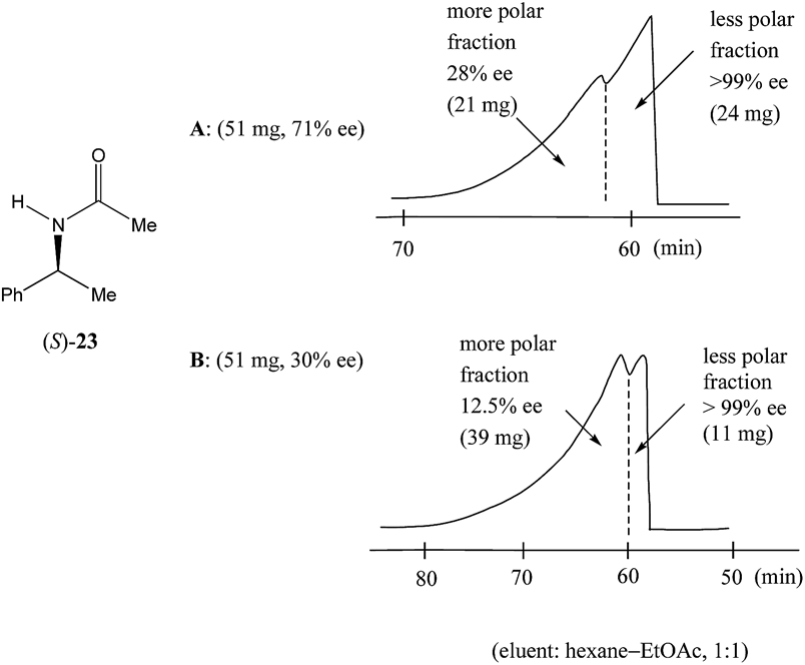
The SDE *via* MPLC of *N*-acetyl 1-phenyl ethylamine (**23**, 71 and 30% ee) using an achiral silica gel column (eluent: hexane–EtOAc, 1 : 1).

Nonetheless, Kitagawa *et al.* systematically studied^11^ the broad application of SDE *via* MPLC to amides of chiral phenyl ethylamines covering various amines {phenyl, *p*-methyl, and *p*-methoxy as well as β-(naphthyl)ethylamine and phenylalanine ethyl ester}, carboxy groups (formyl, acetyl, propanoyl, trifluoroacetyl, benzoyl, *etc.*), and range of initial ee's (30–74% ee). For example, as shown in Fig. 4, the MPLC of scalemic *N*-acetyl 1-phenyl ethylamine (**23**, 71% ee, 51 mg) provided a chromatogram displaying a clear boundary between two fractions of the sample, as would be the case for two chemically distinct compounds. Analysis of the less polar fraction revealed that it contained enantiopure **23** (>99% ee, 24 mg), while the more polar fraction consisted of considerably enantiodepleted **23** (28% ee, 21 mg).

This example is remarkably illustrative for the following reasons. Firstly, amide **23** is one of the simplest chiral compounds lacking any special or unusual structural features. It therefore convincingly suggests that SDE *via* achiral MPLC might be considered as an ordinary, ubiquitous event anticipatable for virtually all scalemic compounds. Secondly, the 24 mg of enantiopure material collected constituted an astonishing 66% yield of the excess enantiomer from the original 51 mg sample of 71% ee, *i.e.* this is the SDE yield. It has to be conceded that fractional crystallization might struggle to attain this level of isolation of the excess enantiomer from such a sample. Thirdly, the MPLC of a sample of 51 mg of **23** of relatively low enantiopurity, 30% ee, also exhibited a boundary between enantiopure and racemic fractions allowing the unconstrained collection of 11 mg of enantiopure (>99% ee) **23**. Very similar trends in most cases were observed for the other compounds examined and altogether, nine of the fourteen compounds examined furnished enantiopure samples (>99% ee) with eight of these providing SDE yields of 47–78% starting from ee's of 48–71% ee prior to the chromatography. It was posited that the formation of syndiotactic heterochiral associations based on amide hydrogen bonds is strongly preferred in the case of these compounds and that these strongly preferred associations were thus responsible for the large magnitude of the SDE.

**Fig. 5:**
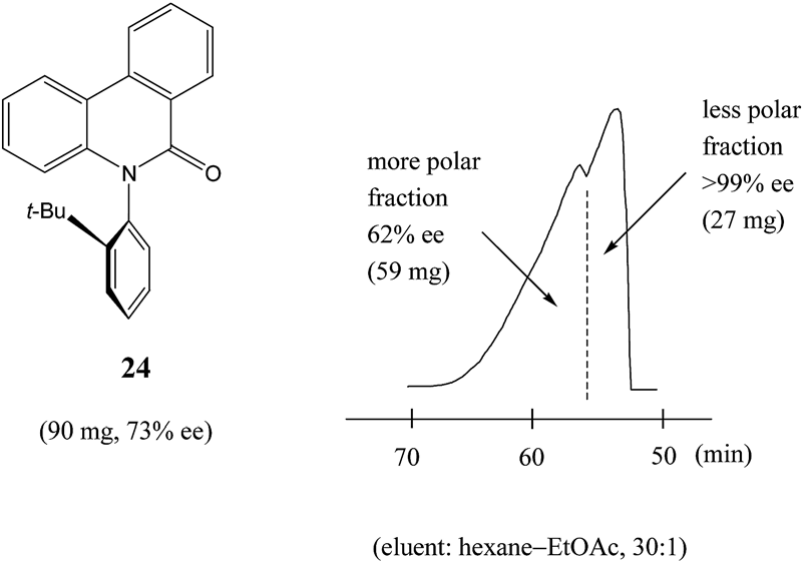
The SDE *via* MPLC of an axially chiral compound, the phenanthridin-6-one derivative **24** (73% ee), using an achiral silica gel column (eluent: hexane–EtOAc, 30 : 1).

Similarly to *N*-acetyl 1-phenyl ethylamine (**23**), a 90 mg sample of a phenanthridin-6-one derivative **24** of 73% ee also displayed13*a* a clear boundary separation (Fig. 5) between fractions containing enantiopure material and fractions containing more racemic material in comparison to the initial sample ee when subjected to MPLC using an achiral silica gel column. Thus, starting from 90 mg of enantioenriched sample of 73% ee, it was possible to obtain 27 mg (41% SDE yield) of enantiopure **24** with an efficiency unmatched by other methods. Similar results were obtained for the compound bearing an isopropyl group instead of a *tert*-butyl group. It should be noted that the MPLC conditions applied for *N*-acetyl 1-phenyl ethylamine (**23**) and the phenanthridin-6-one derivative **24** were not too dissimilar, underscoring the generality and reliability of this approach. Furthermore, compound **24** is chiral by virtue of axial chirality thereby demonstrating that the SDE phenomenon is relevant for other types of chirality as well and not limited to just those possessing an asymmetric center.

In comparison to other chromatographic techniques, the application of MPLC for the detection of SDE and its practical applications has several advantages, including simplicity in the experimental optimization (the ratio of hexane to EtOAc), the presence of usually a clearly visible boundary between enantiopure and more racemic fractions, and the overall cost and practicality in the preparation of enantiopure samples even for samples of initial ee's as low as 30% ee. In fact, MPLC is perhaps one of the finest examples of SDE *via* chromatography and represents one of the best opportunities for exploiting the phenomenon for enantiopurification means. All these factors bode well for the general application of MPLC for comprehensive studies into the SDE phenomenon and represent an opportunity for the application of MPLC as an unconventional enantiopurification method. Indeed, Kitagawa has found MPLC to be a highly useful and routine methodology to effect practical enantiopurification of scalemates resulting from catalyzed asymmetric reactions, *e.g.* in addition to the aforementioned case, ref. 12, 13*b*, 14 and 59. However, if workers are ignorant of the SDE phenomenon and fail to recognize what is happening, they may be enticed into the presumption that the observed splitting of the peak was due to an impurity and thus be tempted to fractionate the peak, and even discard the collected minor component if they do not test it analytically. One can only speculate how many times this may have happened during the course of natural products or asymmetric synthesis work.

#### 3.3.3 Flash

**Fig. 6:**
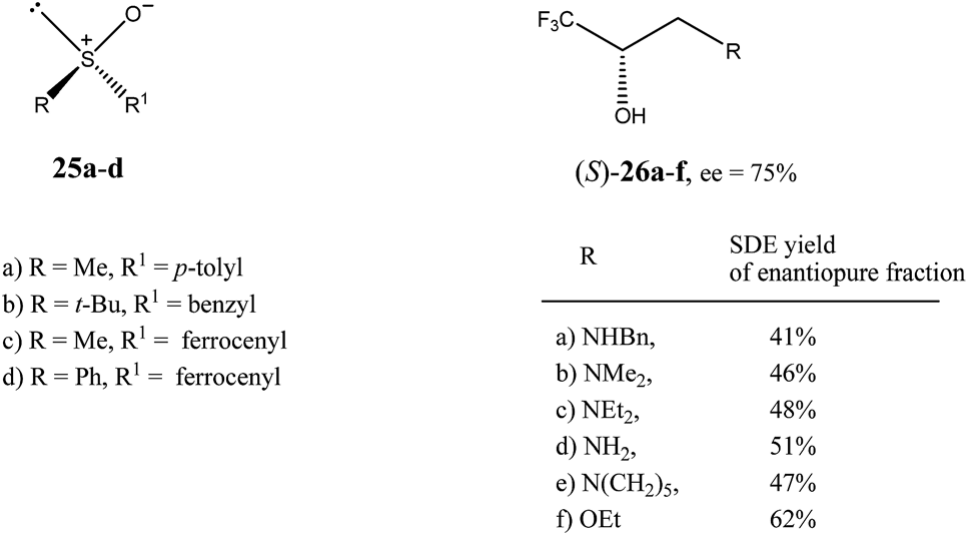
Structures of sulfoxides **25**^16^ and trifluoromethyl-containing secondary alcohols **26**19*b* showing high SDE magnitude by SDE *via* flash chromatography.

The first example of SDE *via* flash chromatography over silica gel was described by Kagan *et al.*^16^ for sulfoxides **25a–d** (Fig. 6). During studies on asymmetric oxidation of prochiral sulfides, the researchers encountered problems with the reproducibility of the stereochemical results. They used flash chromatography over silica gel for the purification of the resulting scalemic sulfoxides, mainly separation from unreacted sulfides and the over-oxidation byproducts, the corresponding sulfones. Further studies and detailed analysis using chiral HPLC of all collected fractions revealed that the ee of the sulfoxides in each fraction varied. For example, the flash chromatography of (*R*)-*p*-tolyl methyl sulfoxide (**25a**) with initial 86% ee afforded 14 fractions in which the ee of the sulfoxide gradually decreased from 99.5% ee in the first fraction to 73.5% ee in the final fraction. The SDE was also observed when either alumina or reverse-phase silica was used as the stationary phase. Other sulfoxides studied in this work, such as benzyl *tert*-butyl (**25b**), ferrocenyl methyl (**25c**), and ferrocenyl phenyl (**25d**) sulfoxides also exhibited SDE *via* flash chromatography over silica gel, showing the generality of this phenomenon for the compounds possessing a sulfoxide group.

The practical aspect of the SDE *via* flash chromatography as a useful method for enantiopurification of the compounds was exemplified by secondary and tertiary alcohols having a trifluoromethyl group directly bound to a stereogenic center.^19^ For example, the flash chromatography of the crystalline or liquid alcohols **26a–f** of initial 75% ee with the three component eluent *c*-hexane–benzene–di-*tert*-butyl ether (1 : 1 : 0.1) in all cases afforded19*b* the *S* enantiomer enantiopure in high SDE yields ranging from 41–62% (Fig. 6). By comparison, the triple recrystallization of a sample of **26a** of 75% ee from ether–hexane provided the *S* enantiomer enantiopure in only 42% SDE yield.

These results clearly demonstrate that SDE *via* achiral chromatography is a simple and effective method for the enantiopurification of scalemic samples that can be also successfully used for liquid compounds, and can be comparable, or even superior, to conventional recrystallization in the case of crystalline compounds. Thus SDE *via* achiral flash chromatography is a good example of the dichotomic nature of the SDE phenomenon, presenting an undesirable complication in the determination of the stereochemical outcomes of asymmetric reactions on the one hand, and, on the other, serving as unconventional yet very general and efficient method for enantiopurification.

#### 3.3.4 Gravity-driven column chromatography

**Fig. 7:**
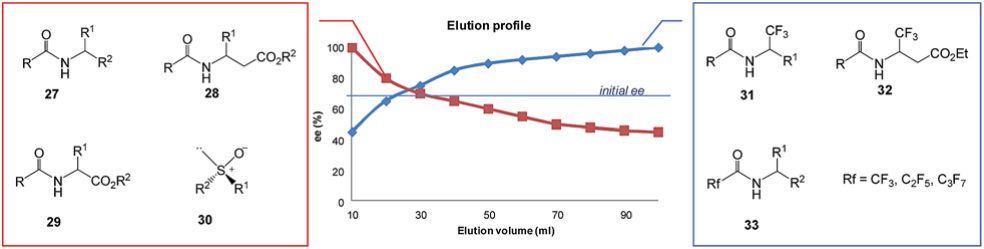
Gravity-driven column chromatography over silica gel for *N*-acetylated amines **27**,^7^–^9^ *N*-acetyl β-amino acid esters **28**,^15^ and *N*-acetyl α-amino acid esters **29**6d,^9^,^11^ as well as sulfoxides **30**6e,^17^,^18^ and perfluoroalkyl-containing compounds such as **31–33**.^4^,^10^

Although gravity-driven column chromatography is a less effective method in comparison to HPLC, MPLC, or flash chromatography, several classes of organic compounds with various functional groups and also different types of chirality have exhibited a strong magnitude of the SDE *via* gravity-driven column chromatography confirming the generality of the phenomenon by chromatography.6*b* Included are compounds containing an amide bond, such as *N*-acetylated amines **27**,^7^–^9^ *N*-acetyl β-amino acid esters **28**,^15^ and *N*-acetyl α-amino acid esters **29**6d,^9^,^11^ as well as sulfoxides **30**6e,^17^,^18^ and perfluoroalkyl-containing compounds such as **31–33**^4^,^10^ (Fig. 7).

All amides **27** derived from 1-phenyl ethylamine underwent the SDE *via* chromatography for a wide range of starting ee's and using different eluent systems.^9^ However, the magnitude of the SDE depended on the steric and electronic properties of the substituents in the acyl group^7^ with the strongest SDE being observed for *N*-acetyl 1-phenyl ethylamine (**23**).^9^ Under optimal chromatographic conditions with regards to enhancement of the SDE, *viz.* using *c*-hexane–methyl *t*-butyl ether as eluent and a substrate loading of 1 mmol of amide **23** per 30 g of gel silica, column chromatography of a sample with an initial 70.0% ee provided an enantiopure fraction (>99% ee) with an 8.7% SDE yield. Column chromatography under the same conditions with samples of higher initial ee, 78.9 and 90.5% ee, afforded enantiopure fractions in much higher SDE yields, 24 and 80.3%, respectively. It should be noted that an enantiopure fraction was also obtained from the chromatography of a sample of lower initial ee, *viz.* 29.6% ee.^8^ The strong magnitude of the SDE observed for amides *via* achiral column chromatography is likely a result of the formation of homo- and/or heterochiral dimers or higher-order species by the formation of hydrogen bonds and molecular calculations that were conducted for *N*-acetyl 1-phenyl ethylamine (**23**) have revealed different stabilities for the homo- and heterochiral dimers.^8^

Similarly good results were obtained for a series of *N*-acetyl β-amino acid ethyl esters using the same eluent system but with a slightly decreased substrate loading (from 30 g to 40 g per mmol of compound).^15^ Depending on the nature of the substituent in the phenyl ring, column chromatography of samples with an initial ∼70% ee delivered enantiopure fractions in 19–46% SDE yields. Chromatography under the same conditions for *N*-acetyl β-phenyl alanine ethyl ester of 94.4% ee provided enantiopure material in 75% SDE yield. Moreover, the column chromatography of *N*-acetylated α-amino acid esters derived from alanine, valine, and phenylalanine also revealed SDE effects for these compounds though the SDE magnitudes were moderately lower.^9^ The chiral elution profiles – the plot of the ee of each fraction *vs.* the fraction or elution volume of each fraction – for all these amides were similar clearly displaying the depreciation in ee from the early fractions enantioenriched in comparison to the initial ee of the samples to the later enantiodepleted ones. In some cases, the use of alumina as the stationary phase resulted in elution order reversal.^15^ These results resoundingly confirm that routine column chromatography can be a fast and convenient method for the enantiopurification of this class of organic compounds.

An interesting result was obtained for amide compounds with a strongly electronegative trifluoromethyl group directly bound to the stereogenic center (*e.g.***31** and **32**)^20^ or a perfluoroalkyl group bound to a carbonyl group (*e.g.***33**).^10^ In both cases, the presence of the strongly electronegative substituent drastically altered the mode of molecular association under chromatographic conditions, and as a result, the opposite elution profile was observed – the first collected fractions were enantiodepleted, while the later ones were enantioenriched (up to 99% ee). It is worth noting that in the case of compounds **31–33**, chlorinated solvents decreased the magnitude of the SDE, *e.g.*, for **31** and **32** a significant reduction of the SDE was observed with CHCl_3_ (ref. 20) while **33** exhibited much smaller SDE when CH_2_Cl_2_ was present in the eluent system. Moreover, in the case of **33**, a reversal of the elution order was observed with CH_2_Cl_2_ indicating that the mode of association preference can be affected by the eluent used.^10^

Another class of organic compounds with a strong tendency for SDE occurrence during gravity-driven column chromatography are the sulfoxides **30**.6e,^17^,^18^ In this case, the driving force for the formation of homo- and/or heterochiral associates necessary for occurrence of the SDE is, in the absence of the possibility for hydrogen bonding, the strong dipole–dipole interaction between the sulfoxide groups. The chromatographic experiments performed^17^ with methyl *n*-pentyl sulfoxide as a model compound as well as with prazoles^18^ (see Section 5.3 The SDE phenomenon and drugs) confirm the great effectiveness of gravity-driven column chromatography for the enantiopurification of sulfoxides. The great advantages of this method is that it can be also be applied to liquid compounds. For methyl *n*-pentyl sulfoxide, it was possible to obtain enantiopure fractions using a sample of very low initial ee, *viz.* 32% ee. Similarly to amides, column chromatography of samples of higher initial ee afforded enantiopure fractions in higher SDE yields. The most optimal chromatographic conditions found for methyl *n*-pentyl sulfoxide utilized aprotic polar solvents such as ethyl acetate as eluent with a reduction of the solvent polarity by the addition of *c*-hexane enhancing the SDE and substrate loading of 1 mmol of sulfoxide per 30 g of silica gel. A decrease in the magnitude of the SDE resulted when using a mixture of acetone and *c*-hexane as eluent or with the addition of a small amount (7.7%) of methanol to the eluent system. A further significant reduction of the SDE, as well as a reversal of elution order, was observed when using alumina as the stationary phase.^17^

Other compounds for which SDE *via* gravity-driven column chromatography has been observed include the mebroqualones,^60^ mandelic acid,^61^ and stilbene oxide,^61^ thus demonstrating that the SDE can be driven by different intermolecular forces. But due to the innate ability of the amides of chiral amines,^7^–^14^ α-amino acid esters,6b,^9^,^11^ and β-amino acid esters^15^ as well as sulfoxides^16^–^18^,^28^ and trifluoromethyl-containing compounds^4^,^19^–^22^ for the formation of homo- and/or heterochiral aggregates by way of strong intermolecular forces resulting in a high magnitude of SDE *via* chromatography, the term SDE-phoric groups has recently been introduced^7^ and applied to these groups (see Section 4.1 SDE-phoric groups and predictability). Clearly from these results, simple column chromatography over silica gel as routinely used for the purification and separation of organic compounds represents a new and unconventional method for the enantiopurification of either crystalline or liquid compounds owing to the possibility of the SDE phenomenon occurring. The amenability to liquid compounds is a considerable advantage over crystallization which is limited to only crystalline samples. On the other hand, chromatographic purification may also potentially create problems for the correct determination of the stereochemical outcomes of asymmetric syntheses when column chromatography is employed in the work-up of reactions.^62^–^64^ For this reason it is strongly recommended that researchers conduct a test^65^ for the occurrence of SDE to ensure that it does not affect the true stereochemical results of asymmetric reactions. Similar caveats of course also apply to natural products and all other areas involving chiral-based studies.

#### 3.3.5 SEC

**Fig. 8:**
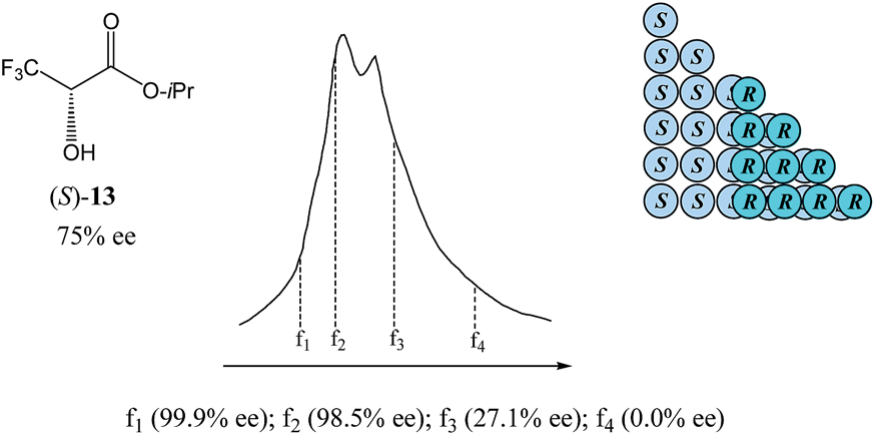
The SDE *via* SEC of a sample of isopropyl 3,3,3-trifluorolactate (**13**) of 75% ee exhibiting a distinct boundary within the elution profile intimidating two peaks and resulting in both enantiopure and racemic fractions being obtained.

Size-exclusion chromatography (SEC), also referred to as molecular-sieve chromatography, differs from chromatography *per se* in the usual sense as it does not, in the main, rely on a physicochemical process, *viz.* sorption–desorption, for the discrimination of analytes, but rather a purely physical one, *viz.* the ability of the analytes to diffuse into the pores of the stationary phase and be retained, thus retarding their elution. This inclusion–exclusion process by elution through a gel is thus used for the separation of molecules based on their size, and in some cases molecular weight, since small molecules readily diffuse into the pores resulting in an increased retention time, while large molecules, less retained by the pores, are eluted in a shorter time.^66^ While SEC is applied almost exclusively for the separation and purification of large molecules such as proteins or polymers, or in natural products work for the initial separation of small molecules from the matrix of large biomolecules, there is no reason this technique cannot be applied more widely to small, organic molecules. Moreover, considering that the dynamic formation of monomers *vs.* homo- and heterochiral higher-order species – and thus resulting in species of different size and molecular weight – is the principal cause of SDE *via* achiral chromatography, it can be envisaged that SEC could be the most suitable technique for the exploration of the SDE phenomenon and that SEC should be supremely amenable to the practical application of the SDE phenomenon for enantiopurification purposes. However, SDE *via* SEC, along with SDE *via* GC (see Section 3.3.6 GC), is the least studied of the major chromatographic techniques and remains a virtually unexplored area of research, indeed, there are only two known reports on SDE *via* SEC. While the first report^67^ concerns some relatively large tetrapeptide molecules, the second report^51^ concerning isopropyl 3,3,3-trifluorolactate (**13**, Fig. 8) reveals the exciting potential of SEC as it is applied to regular-sized organic molecules. Both reports on SDE *via* SEC, it is worth noting, strongly support the conjecture of the dynamic formation of monomers *vs.* homo- and heterochiral higher-order species.

As can be seen in Fig. 8, the SEC of a sample of **13** of 75% ee revealed a distinct boundary within the elution profile intimidating two peaks, within which are contained enantiopure and racemic fractions in the first eluting and second eluting “peaks”, respectively – akin to what has also been observed in MPLC (see Section 3.3.2 MPLC). Accordingly,^51^**13** shows the “ultimate preference for homochiral intermolecular interactions” by forming hydrogen bond-based chains in the solid state as well as in solution.^49^

The size of the higher-order species in solution can be estimated^68^ as ranging from dimers to decamers and the homochiral oligomers are syndiotactic, adopting alternating orientations of **13** along the chain to avoid electrostatic repulsive interactions between the trifluoromethyl groups.50b,^69^,^70^ Hence, the first fraction *f*_1_ was found to consist of enantiopure **13**, followed by the significantly enantioenriched fractions *f*_2_ and *f*_3_, and remarkably, the last eluted fraction *f*_4_ was shown to be racemic, thereby allowing for the unprecedented preparation of both enantiopure and racemic forms of **13** in one simple procedure. While this unique preparative attribute might be purely serendipitous, the potential for SEC to have an apparent advantage over other chromatographic techniques is palpable. Since for SEC the elution of higher-order species over monomers is always absolute, it offers enormous benefit in mechanistic elucidations and therefore, quite rational application of the SDE *via* SEC.

#### 3.3.6 GC

**Fig. 9:**
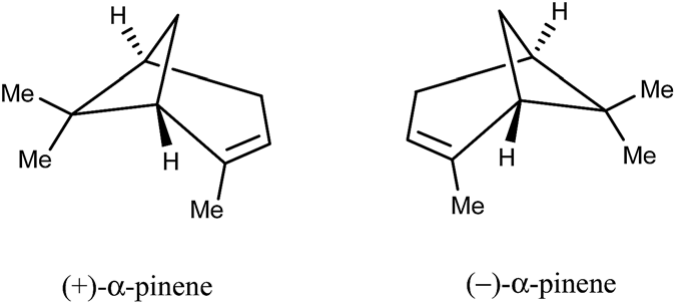
Structures of the enantiomers of α-pinene (**34**).

There is only one report^71^ of the SDE phenomenon occurring by way of GC wherein the elution behavior of the hydrocarbon α-pinene (**34**, Fig. 9) on a non-polar capillary GC column was described. While the results and interpretation are credible, unfortunately the “gold standard” was not applied, *viz.* measurement of the ee across the eluting peak to verify that the ee varies as per the SDE phenomenon. The authors ascribed the behavior of **34** to “dynamic modification of the stationary phase”, which can be taken as analyte association within the liquid stationary phase. Furthermore, the workers also presented convincing arguments why SDE *via* GC might be difficult to observe – that the concentrations required to observe the phenomenon in most cases start to overlap with the limits of column overload which then mask the SDE effect due to their opposing effects on the peak profile and elution. Moreover, this constraint means that results can be difficult to replicate by other workers in cases where the SDE *via* GC phenomenon has been observed unless absolutely identical conditions are applied, which is near impossible in practice with hypersensitivities to such variables as the condition of the column.

In addition, the authors also alluded to possibility of SDE *via* GC having occurred in several other instances from consideration of a database consisting of 12 000 cases, though it is unclear how many of these cases involve, not only chiral compounds, but specifically scalemic samples. Nevertheless, it can be taken that the occurrence of SDE *via* GC is likely to be an extremely rare event. On a practical level, this implies that there is likely to be little danger of errors occurring due to SDE *via* GC, especially given the difficulty of even effecting the SDE *via* GC deliberately due to the overlapping limits imparted by column overload. Thus SDE *via* GC is unlikely to represent an opportunity, and concurrently, unlikely to represent a menace except in the most exceptional of circumstances and it represents just an event of novelty value for workers at large. Hence SDE *via* GC can be considered benign and of research interest only to a select band of specialists as SDE *via* GC, along with SDE *via* SEC (see Section 3.3.5 SEC), is the least studied of the major chromatographic techniques and remains a virtually unexplored area of research. And although SDE *via* GC remains to be unequivocally proven, it is not outside the realm of possibility given that SDE *via* distillation has been demonstrated (*vide supra*). Perhaps an alternative candidate to test for SDE *via* GC is isopropyl 3,3,3-trifluorolactate (**13**, Fig. 1), one of the compounds that has been shown to undergo SDE *via* distillation.

## 4 Utilization of the SDE phenomenon

### 4.1 SDE-phoric groups and predictability

While the realization has developed that one should expect all chiral compounds to possibly exhibit some degree of SDE under certain conditions, the phenomenon, in principle at least, always occurs whenever any physicochemical process is applied to a scalemic sample, though it may be vanishingly small in many instances and the observed magnitude can vary dramatically depending on a compound's structure and the prevailing conditions. However, the concept of SDE-phoric groups^7^ is a recently introduced concept that has the potential to be very useful in terms of pedagogy, prediction, and the facility to alert workers to the possibility of the occurrence of the SDE phenomenon for the compounds with which they are working, both from the point of view of being deleterious to their results but also with respect to beneficial possibilities by providing a novel means of enantiopurification. The concept of SDE-phoric groups is that the presence of SDE-phoric groups in a molecule means that such compounds are likely to have a propensity to exhibit a strong magnitude of the SDE, a property imparted on them by the SDE-phoric groups. And not only is the magnitude of the SDE likely to be greater for such molecules, the observable occurrence of the SDE is likely to be more persistent over a wider variety of applied conditions. Groups identified as SDE-phoric include the amides of chiral amines,^7^–^14^ α-amino acid esters,6b,^9^,^11^ and β-amino acid esters^15^ as well as sulfoxides^16^–^18^ and compounds containing a trifluoromethyl group.^4^,^19^–^22^ Paradoxically, SDE-phoric groups can even cause the magnitude of the SDE to diminish, even to the point of it seeming to disappear altogether. But this seeming paradox can be resolved since one of the resultant effects that SDE-phoric groups impart on molecules is to alter drastically the Δ*E* between the homo- and heterochiral associates. A large Δ*E* can be associated with a strong magnitude of the SDE. Thus if Δ*E* is small, an SDE-phoric group will result in an enhanced Δ*E*. But if Δ*E* is already large, then an SDE-phoric group may enlarge it even further, or conversely, reduce it by a substantial amount thus leading to a reduction in the magnitude of the SDE. Trifluoromethyl groups are especially amenable to possessing this particular trait.

In addition to qualitative descriptions, quantification of SDE occurrences has begun, *e.g.* the magnitude of the SDE^8^ (eqn (1)), the SDE range (eqn (2)), and the SDE yield (eqn (4)), and while prediction of the molecules with respect to expected results has started with the concept of SDE-phoric groups,^7^ prediction of the quantification of the results and under what conditions the SDE will occur remains the challenge. Nevertheless, in terms of suppressing the SDE when it is observed or to try to ensure that it is less likely to occur in high magnitude, this can be accomplished by introducing interfering or competing solvent–solute interactions, *e.g.* incorporating protic solvents when the formation of analyte associates is hydrogen-bond based or to simply reduce the concentration of the analyte. Alternatively, to accentuate the SDE, then such interfering or competing solvent–solute interactions should be limited, and/or the concentration of the analyte increased, and/or the temperature of the system (if possible) decreased to increase the amount of intermolecular association.

Other great challenges are to predict the direction of the SDE, *e.g.* for SDE *via* chromatography, does the racemic portion or the excess enantiomer portion elute first or last, and to model precisely the SDE behavior with respect to minor aberrations (or at least explain them). The direction and even the magnitude of the SDE may be possible from theoretical calculations (by modeling the energies of the associates^8^,^24^,^25^) and from experimental observations such as NMR^25^ and other spectroscopic methods.24*d* Thus, while it remains the case that one can never be sure that results have not been unduly altered by the SDE, the need to perform SDE tests for detecting the occurrence of the SDE phenomena is paramount.^65^

### 4.2 New directions and novel, unconventional enantiopurification methods

From the foregoing sections, it can be readily ascertained that SDE by various means other than recrystallization (*e.g. via* chromatography, especially MPLC) is a practical and fully implementable means for enantiopurification. Indeed, SDE *via* chromatography has even been claimed to be comparable, or even superior to, recrystallization as a means to obtain enantiopure material with respect to SDE yield. In regards to comparing the capabilities of recrystallization *vs.* SDE *via* chromatography, there are three cases to consider. For conglomerates, comprising 5–10% of compounds,^23^ there is little or no advantage in theory either way and in principle, essentially all of the excess enantiomer can be isolated from a scalemic mixture by either process (so *Y*_SDE_ = 100%), though with kinetic effects the thermodynamic limit can be exceeded in recrystallization. For solid solutions, no gain in enantiopurification can be effected by recrystallization and SDE *via* chromatography holds a considerable advantage for these quite rare cases.^23^ For racemic compounds, which comprise the vast majority of compounds,^23^ it is not possible to compare in a simple way the theoretical SDE yields of the pure enantiomer from a scalemate applying SDE *via* chromatography *vs.* recrystallization. The former is in principle dependent only on the ee and the theoretical SDE yield approaches the ee (so *Y*_SDE_ = 100%) in the limit of the process (eqn (3)) while the latter is dependent on both the ee and the eutectic point (ep). The maximum theoretical yield by recrystallization (*Y*_max,SvR_) for racemic compounds is given by the formula (re-written in terms of ee from the formula taken from ref. 23*b*):

5 *Y*_max,SvR_ = (0.5ee – ep + 50)/(100 – ep) × 100

where ep is expressed as a mole fraction percentage.

Thus, *Y*_max,SvR_ increases with the ee for a given ep and tends to 100% in the limit; with increasing ep, *Y*_max,SvR_ is reduced for a given ee. On the other hand, if the maximum theoretical yield by SDE *via* chromatography (*Y*_max,SvC_) is taken as the ee (eqn (3)) since in the limit it tends to this value, then it too tends to 100% with increasing ee obviously. With increasing ee, both *Y*_max,SvR_ and *Y*_max,SvC_ tend to the same limiting value and are not differentiated significantly at high ee. As the ep tends to the other extreme, 0% ee (*i.e.* the conglomerate minimum), both the *Y*_max,SvR_ and *Y*_max,SvC_ converge to the same terminal value (the ee) and hence they again converge towards parity. Table 1 illustrates the dependency of *Y*_max,SvR_ on ep and ee.

**Table 1:** The dependency of *Y*_max,SvR_ on ep and ee

No.	ep	ee	*Y*_max,SvR_
1	50^*a*^	1	1.0
2	50	70	70.1
3	50	90	90.0
4	60	21	1.3
5	60	30	12.5
6	60	50	37.5
7	60	70	62.5
8	60	90	87.5
9	60	98	97.5
10	75	51	2.0
11	75	70	40.0
12	75	90	80.0
13	75	98	96.0
14	90	81	5.0
15	90	90	50.0
16	90	98	90.0

^*a*^Note that an ep of 50% equates to conglomerate behavior.

In effect therefore, SDE *via* chromatography is favored over recrystallization – kinetic effects in the recrystallization process aside – but since both tend to 100% as the ee approaches 100% and the two become equitable, the preference diminishes at high ee's and similarly at lower ep's where they both tend to the ee. In short, the likely preference for SDE *via* chromatography over recrystallization is accentuated by either lower ee's or higher ep's.

Aside from theoretical considerations of SDE yield, there are also practical aspects that come into play. For recrystallization, the right solvent system needs to be found by repetitive testing, and of course the material obviously needs to be crystalline or otherwise crystalline derivatives need to be prepared – in which case it would be more sensible to prepare diastereomeric derivatives using a chiral derivatizing agent (CDA). Clearly substantial additional work can be involved. SDE *via* chromatography thus holds a considerable advantage over recrystallization since crystalline material is not required. Furthermore, the ep of a compound is not known *a priori*^72^ whereas SDE *via* chromatography is a relatively controllable process, *e.g.* the concentration of the analyte can be simply increased to increase the intermolecular associations, the solvent can be easily altered to manipulate the elution time or to minimize interfering or competing solvent–solute interactions, *etc.*

But despite the long history of crystallization, even now new crystallization methodologies are being developed to effect the SDE. For example, it has recently been reported^73^ that gas antisolvent fractionation (GASF) using carbon dioxide caused SDE *via* precipitation of *ortho*, *meta*, and *para*-substituted chlorinated mandelic acid scalemates from acetonitrile solution. The behavior in terms of enantiopurification was very similar to the recrystallization of racemic compounds under thermodynamic control. The main advantages of the method are speed and the economical use of organic solvent, not to mention the capability of scaling the process up to an industrial scale-size.

Distillation would also be an attractive option as a means for the enantiopurification of bulk material that could be applied on an industrial scale akin to crystallization and sublimation if not for the limitation that the number of chiral compounds likely to show SDE *via* distillation is small, let alone be sufficiently volatile to be amenable to practical distillation in the first place. But other means to process scalemic samples on a practical or industrial scale in addition to the well known processes of crystallization and sublimation are possible, such as force field (see Section 3.1 SDE *via* force field). Another potential large-scale process is foam fractionation,^74^ though there have never been any reports of SDE *via* foam fractionation thus far. Of course there are inherent dangers associated with any such process that can lead unwittingly to modification of the ee of the fractions, even on the industrial scale, if due care is not taken. And although the application of distillation for practical purposes obviously might be of extremely limited use, the use of chiral selectors (CS) to form diastereoazeotropes as a means of resolution has been touted, though the principle has yet to be demonstrated in practice.^75^ However, the use of a CS to effect enantiodifferentiation has been demonstrated in the case of foam fractionation.^76^

**Fig. 10:**
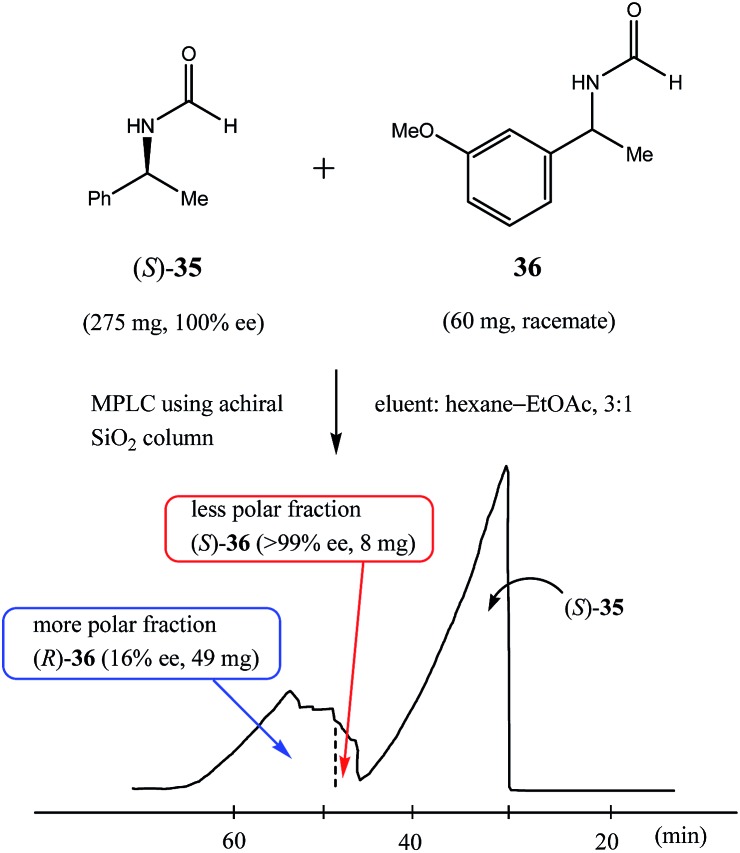
The pseudo-SDE (chiral selector-assisted SDE resolution of racemates) of (*rac*)-*N*-formyl-1-(3-methoxy)phenyl ethylamine (**36**) using (*S*)-*N*-formyl-1-phenyl ethylamine (**35**) as the CS by MPLC equipped with an achiral silica gel column (eluent: hexane–EtOAc, 3 : 1; ratio of CS : substrate, 5.5 : 1).

Along these same lines is the concept of the pseudo-SDE (chiral selector-assisted SDE resolution of racemates) process which has been successfully demonstrated^77^ and is much more applicable to laboratory-scale methods. The process effectively mimics or simulates genuine SDE *via* chromatography, but using a CS to effect the enantiodifferentiation instead of the excess enantiomer. The trick is to have a closely eluting, structurally similar CS to the substrate. Thus, enantiopure (*S*)-*N*-formyl-1-phenyl ethylamine (**35**), in addition to other amides tested, was used^77^ to obtain enantiopure samples (>98% ee) by MPLC of various racemic *N*-formyl-1-aryl ethylamine derivatives – eleven successfully from the thirteen racemates tested with yields of 14–28% using a ratio of 5.5 : 1 of CS to substrate. Interestingly, not in all cases did the CS co-elute (fully or only partially) with the substrate, but the early association between the CS and the substrate on the column was sufficient to effect enantiodifferentiation where co-elution did not occur, for example, see Fig. 10 for the enantiodifferentiation by pseudo-SDE of (*rac*)-*N*-formyl-1-(3-methoxy)phenyl ethylamine (**36**). The significance from an SDE perspective of pseudo-SDE is that an indication of the elution order between dimeric associates and monomers can be inferred as well as the preference between homo- and heterochiral associates. For the systems just described, it seems dimeric associates elute faster than the monomers and in each case homochiral associates are favored over heterochiral associates.

**Fig. 11:**
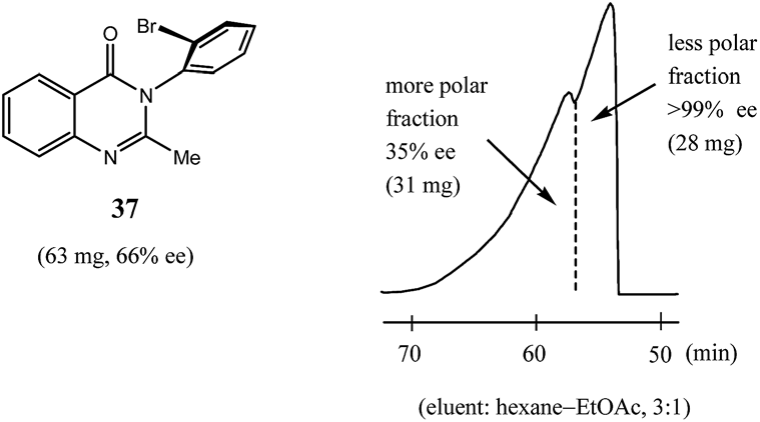
The SDE *via* MPLC of mebroqualone (**37**, 66% ee) using an achiral silica gel column (eluent: hexane–EtOAc, 3 : 1).

Finally, while dipole–dipole and aromatic π–π interactions, and hydrogen bonds especially, are well recognized as interactions that can give rise to the SDE phenomenon, halogen bond-driven SDE was an unexpected observation,^60^ and moreover, a strong magnitude of the SDE phenomenon was found for compounds where halogen bonding was present. Thus, in the examination^60^ of a set of eight mebroqualone derivatives by both MPLC and gravity-driven column chromatography, in all six cases subjected to MPLC, enantiopure samples (>99% ee) were obtained with Δee's of up to 81% ee and SDE yields generally high, even for samples of initial ee's as low as 28.4% ee and were as much as 74%. Gravity-driven column chromatography provided enantiopure samples (>99% ee) for three of the six compounds with the remaining three compounds tested still yielding samples of >90% ee, all from initial samples of 61–70% ee. Pertinently, similarly to the results reported above for MPLC (Section 3.3.2 MPLC), a clear boundary separation was again evident between the fractions containing enantiopure material and fractions containing more racemic material in comparison to the initial sample ee when samples were subjected to MPLC using an achiral silica gel column. For example, in Fig. 11 is portrayed the results for a 63 mg sample of mebroqualone (**37**) with an initial ee of 66% and which provided an enantiopure fraction of 28 mg, equating to an SDE yield of 68%. Again, it is worth noting that the MPLC conditions applied were not too dissimilar as other reports and only required the adjustment of the ratio of hexane to ethyl acetate in the eluent, thus further underscoring the generality and reliability of this approach.

Considering that halogen bonding interactions can be rationally designed and can match, or even exceed, the strength of the more familiar hydrogen bond, the discovery of halogen bonding interactions being able to effect SDE *via* chromatography clearly opens an unexpected new direction in SDE research. Moreover, compounds containing a halogen and a carbonyl group, compounds not previously considered as particularly prone to the SDE phenomenon, encompass a great number of molecules possessing the potential for halogen bonding to be present. Furthermore, it is also generally considered necessary for very large energy differentials (in comparison of the homo- and heterochiral associates) to be in place for the interactions for the SDE phenomenon to occur. This is actually not the case, *e.g.* for 1,1′-bi-2-naphthol (**16**) only a marginal difference in energies was found.^25^ This is perhaps one notable misconception by workers who are aware of the SDE phenomenon and may be the basis for leading them to think that the SDE phenomenon is a rare occurrence.

## 5 Implications of broader significance

### 5.1 NLE's in asymmetric catalysis

**Fig. 12:**
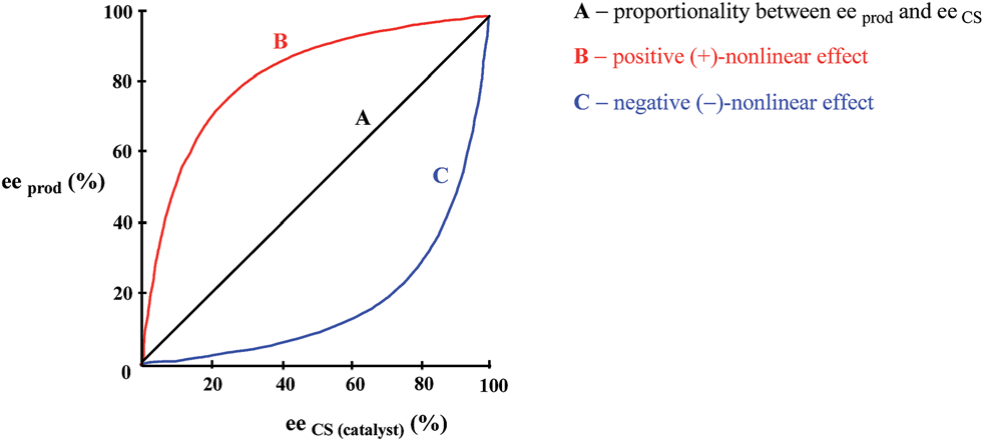
Plot the ee of the product *vs.* the ee of the CS to portray NLE's.

As enunciated by Kagan,78*a* “In many enantioselective reactions ee_prod_ is *not always proportional* to ee_aux_” and such occurrences are known as nonlinear effects (NLE's) in catalytic asymmetric synthesis. Positive NLE's are denoted when the ee of the product is greater than that of the CS (catalyst) and negative NLE's denoted when the ee of the product is less than that of the CS. The conventional manner to express NLE's is to plot the ee of the product *vs.* the ee of the CS (Fig. 12).

Due to their high relevancy to practical organic synthesis, NLE's in catalytic asymmetric syntheses have been meticulously studied, most noticeably by Kagan,^78^ Noyori,^79^ and Soai.^80^ Various models have been developed by Kagan to explain the experimental observations, but the basic commonality to all of them is that homo- and heterochiral associates/complexes are formed. Thus with a scalemic catalyst, or indeed a scalemic starting substrate with a racemic catalyst, this can lead to deviations from direct proportionality between the ee of the catalytic CS and the ee of the product due to the differential formation of homo- and heterochiral higher-order species.78*e* While ML_*n*_ (*n* = 2–4 with L a chiral ligand and M a metal) systems were described by Kagan,78*a* their analysis applies equally well to systems lacking a metal for coordination and based on, for example, hydrogen bonding.^78^ The models developed by Kagan are generally extremely robust in describing observations wherein the ee of the product comes about due to the combination of various factors: thermodynamics determining the composition of monomer and homo- and heterochiral associates (*i.e.* the position of the equilibrium), kinetics determining the reaction rates of each catalytic species (the competition between the catalytically active species whether they be complexes or otherwise to provide more of their product), and the enantioselectivities of each catalytic species (relative between the competing catalytic species to provide more racemic or more enantiopure product). Blackmond^81^ has expanded further on the kinetics of catalytic asymmetric reactions as there are many additional aspects in practice to consider, monomer *vs.* dimeric associates as active catalysts (*i.e.* either monomer or complex or both can be catalytically active), reversible *vs.* irreversible associate formation, stoichiometric reactions, *etc.*^81^ The consummate accomplishment of Kagan was to compress these factors into manageable equations, which, with the input of appropriate parameter values, can be plotted and compared to experimental plots to confirm the validity of the postulated catalytic system for the reaction under study. Another way to view the system is the alternative mathematical model proposed by Kagan, the reservoir model.78*a* This is a conceptually simpler to view the process and which also provides a general solution (as opposed to each ML_*n*_ system having its own unique solution).

**Scheme 5:**
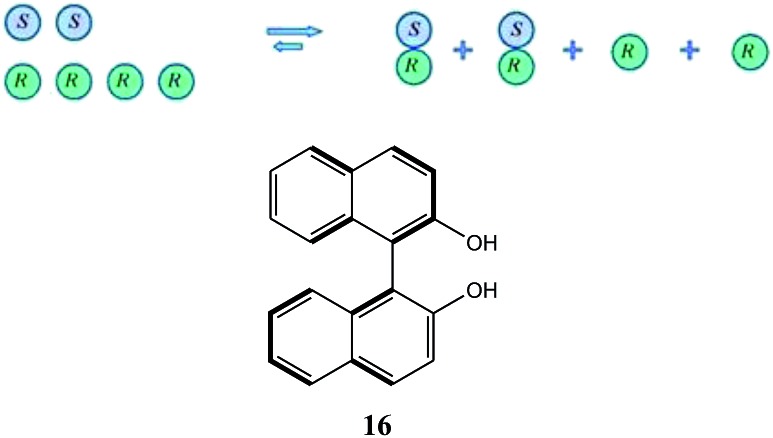
Underlying principle of a positive NLE and the structure of axially chiral 1,1′-bi-2-naphthol (**16**).

To take a simplistic, extreme case, if a catalyst has a tendency towards the formation of heterochiral dimers (Scheme 5), then in a system of the scalemic catalyst there will be an equilibrium between monomeric *R* and *S* enantiomers and the corresponding homo- and heterochiral dimers with a preponderance of the heterochiral dimers over the homochiral dimers leaving an excess of the monomeric *R* enantiomers disproportionate to the overall ee composition of the sample. In cases where the equilibrium is shifted well towards heterochiral dimers and the dimers are inactive catalytically, a very strong positive NLE results as only the free monomeric excess enantiomers catalyze the synthetic transformation providing a stereochemical outcome closer to that provided by the enantiopure catalyst. In the case of negative NLE's, a similar mode of events takes place but with the difference that homochiral species are preferred.

The corresponding homo- and heterochiral dimers, or higher-order species, can also be formed with pseudo-enantiomers, structurally similar molecules, giving rise to the concepts of catalyst activation^82^ and poisoning^83^ in catalytic asymmetric synthesis. This area has also been well researched and fully rationalized mechanistically. Much less appreciated, however, is that these cases of NLE's represent examples of strong SDE magnitude as the chromatography of a mixture represented in Scheme 5 will result in near complete separation of the racemic portion from the excess enantiomer portion and that this can therefore be utilized as an efficient enantiopurification method. This has been strikingly demonstrated for 1,1′-bi-2-naphthol (**16**) which shows strong NLE's^84^ as well as exceptionally strong SDE magnitude under the conditions of achiral chromatography.24c,e,^27^,^54^

Obviously if workers are unaware of NLE's they can assume an incorrect ee of the product, an incorrect mechanism of the reaction, incorrect value for the ee of the catalyst, incorrect enantioselectivity for the catalyst, or even the incorrect enantiomer of the products as the opposite enantiomer preferentially produced by the catalyst can change depending on the ee of the catalyst78*a* (*e.g.* quaternary associates) or stage of the reaction^81^ for certain systems. So errors of both Type I and Type II can be incurred, *i.e.* methods which are purported to give good stereoselectivity but do not generally as well as methodologies which are discarded (or used wrongly for interpretations) due to stereoselectivities which were evaluated as poor but which in fact could be much better than realized. Applications to other systems without regard for NLE's can be fraught with resulting errors. Though problems can potentially arise if workers are unaware of NLE's and do not take into account their vagaries, they can also be highly beneficial. For example by permitting the use of cheaper catalysts of lower enantiopurity to attain comparable results – or even potentially superior results in exceptional though yet to be demonstrated cases78*a* – as expensive, high enantiopurity reagents which can be dispensed with altogether in special cases.78a,^81^ NLE's also provide a means to probe the mechanism of reactions by conforming to one of the postulated mathematical models.78a,^81^ In general, the formation of homo- and heterochiral associates in solution is an inherent property of all chiral organic molecules with the only difference between compounds being the position of the equilibrium between the corresponding monomers and higher-order species. Thus, while application of homo- and heterochiral associates in the area of catalytic asymmetric synthesis has been properly explored, its relevance to the SDE remains underappreciated and overlooked. Indeed, occurrences of NLE's in catalytic asymmetric syntheses are one of the most conspicuous observations of the fundamental SDE mechanism. Moreover, some of the results and predictions rival the incredulity of SDE *via* distillation and there seems no limit to the ability of the SDE phenomenon to surprise.

### 5.2 The SDE phenomenon and drugs

As is well known, the two enantiomers of a chiral drug can have very different physiological effects, and since many pharmaceuticals on the market are chiral, the implications of the SDE in this context are readily perceived. In the extreme, one enantiomer might be beneficial providing the required effect to treat the malady while the other is harmful. Thus many new drugs, and even some older ones originally marketed as racemic material, are now required by law to be sold enantiopure. But the question naturally arises, has the SDE phenomenon been properly considered in the production of chiral drugs?

**Fig. 13:**
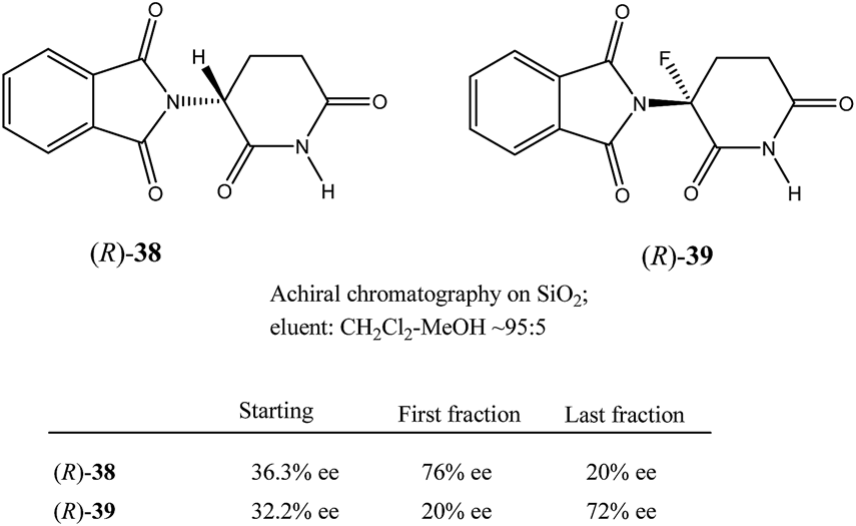
The SDE *via* achiral gravity-driven column chromatography of thalidomide (**38**) and fluorothalidomide (**39**).

The quintessential textbook example of diametrically opposing pharmaceutical attributes is the infamous case of thalidomide (**38**).^85^ And as discovered by Shibata's group,^26^ thalidomide (**38**, Fig. 13) exhibits high magnitude of the SDE *via* achiral chromatography. For example, a sample of thalidomide (**38**), originally of 36.3% ee, when subjected to routine gravity-driven column chromatography over silica gel produced early fractions that were noticeably enantioenriched (>70% ee), while the final fractions were markedly enantiodepleted (down to 20% ee). To overcome the innate rapid racemization of thalidomide (**38**) under physiological conditions which hinders its medicinal applications, many research groups^86^ have prepared various configurationally stable derivatives of thalidomide (**38**). One promising example is fluorothalidomide (**39**) which exhibits high anticancer activity^87^ and possesses a quaternary chiral carbon. While it was found^26^ that fluorothalidomide (**39**) showed a similar level of SDE magnitude under the conditions of commonly used achiral gravity-driven column chromatography, unexpectedly, the order of enantioenriched and -depleted fractions for fluorothalidomide (**39**) was reversed in comparison to thalidomide (**38**), thereby indicating an opposite preference between homo- and heterochiral higher-order species formation in comparison to thalidomide (**38**) under the prescribed conditions. The authors^26^ described the propensity for the SDE *via* chromatography of compounds **38** and **39** as ubiquitous since the SDE was observed under a wide variety of chromatographic conditions, *e.g.* using either regular gravity-driven column or flash chromatography, mesoporous silica gel or alumina as the stationary phase, and various combinations of solvents as eluents.

**Fig. 14:**
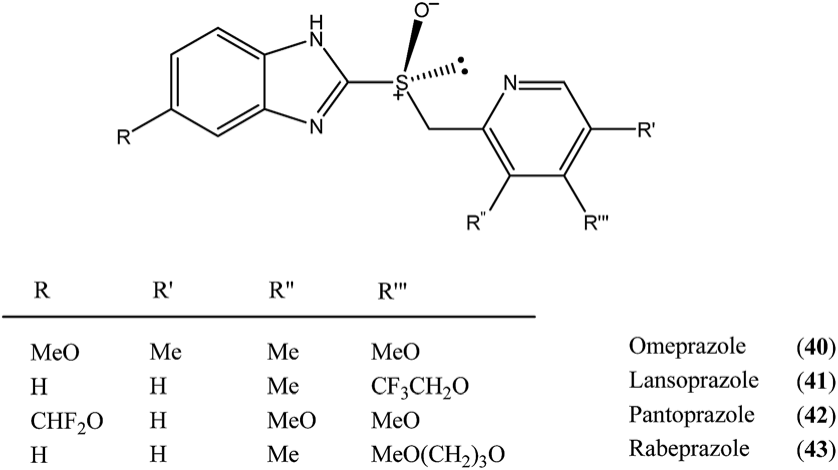
Structures of omeprazole (**40**), lansoprazole (**41**), pantoprazole (**42**), and rabeprazole (**43**).

Other well known drugs exhibiting strong magnitude of the SDE phenomenon are the sulfoxide drugs the prazoles **40–43** (Fig. 14), a family of proton pump inhibitors commonly used for the treatment of peptic ulcers and which are among some of the top-selling drugs in the current pharmaceutical market. SDE experiments with compounds **40–43** were conducted using routine gravity-driven column chromatography over regular silica gel.^18^ In all four cases, the early fractions were noticeably enantioenriched while the later fractions were accordingly enantiodepleted. Of note, the magnitude of the SDE was, surprisingly, not greatly influenced by the ee of the starting samples, which ranged from ∼20 to ∼90% ee, and the Δee was similar for all compounds **40–43**, ∼20% ee. Nonetheless, the authors^18^ emphasized the convenience of preparing enantiopure (>99% ee) samples of prazoles **40–43** starting with material of about 84–88% ee. As pointed out in Section 4.1 SDE-phoric groups and predictability, a sulfoxide group is considered an SDE-phoric group and compounds **40–43** clearly underscore this notion. The rationale^18^ for the observed SDE profiles of prazoles **40–43** is the formation of relatively stable homochiral dimers based on hydrogen bonding between the S–O and the N–H groups, a plausible notion supported by observations both in solution^28^ and in the solid state.^88^

**Fig. 15:**
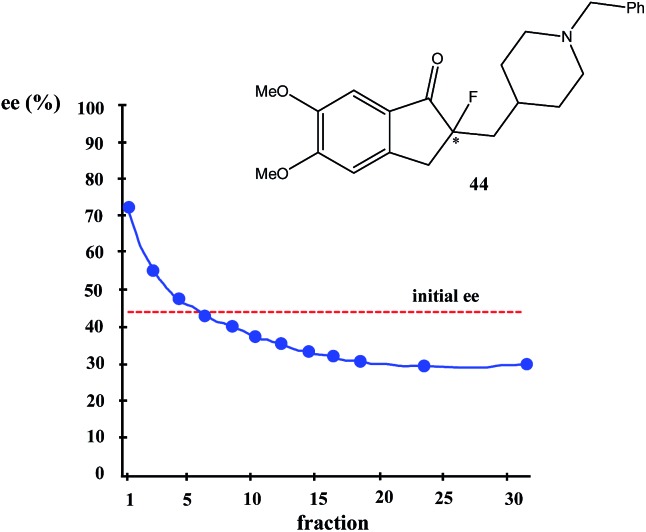
Structure of the fluorinated analog of donepezil (**44**) and the SDE *via* achiral gravity-driven column chromatography of a sample of **44** with 44% ee (eluent: hexane–EtOAc, 1 : 4) showing the ee of collected fractions.

One new, highly promising candidate for the treatment of Alzheimer's disease and other Alzheimer-like diseases is the fluorinated analog of donepezil,^89^**44** (Fig. 15), specifically designed to combat the problem of *in vivo* racemization of donepezil, though there is only a minor difference between the bioactivities of the two enantiomers of donepezil. However, not only is **44** much more potent than donepezil against acetylcholinesterase (1.3 nM *vs.* 5.9 nM), it could be that there are large bioactivity differences between the two enantiomers of **44** based on the 60-fold difference between the two enantiomers of a very similar difluoro analog of donepezil towards rat brain acetylcholinesterase. Thus, Shibata *et al.*, upon noticing a rather minor but unexpected change in the ee when conducting a non-asymmetric transformation of a scalemic sample of **44**, specifically tested^89^ for the SDE when performing gravity-driven column chromatography on a sample of **44** (Fig. 15) with 44% ee and observed a substantially high Δee of 43%.

Other examples of drugs exhibiting SDE are the mebroqualone-type GABAergic drugs possessing axial chirality13b,^14^,^60^ (see Section 4.2 New directions and novel, unconventional enantiopurification methods), cephalotaxine,^90^ precursors to norepinephrine transporter inhibitors,^12^ chloromezanone^54^ (**19**), and benzodiazepine camazepam^54^ (**20**) in addition to many others.^91^ It is worth noting that focused research on the SDE properties of marketed drugs has never been systematically undertaken and all reported examples are the results of accidental or anecdotal observations. Yet, taking into account the generality of the SDE phenomenon, it can be anticipated that all chiral drugs might have measurable magnitudes of the SDE *via* achiral chromatography as well as sublimation in the case of volatile chiral products or intermediates, *e.g.* the drugs ibuprofen^92^ and naproxen.92*a* Thus it is not unreasonable to consider that the storage of highly enantiopure drug material may result in the sublimation {see Section 3.2.2 Solid–gas (sublimation)} of the minute amount of the racemic portion to the higher levels of the containing vessel, as in fact has been observed before,^57^ and any indiscriminate removal of the material for analytical purposes may skew ensuing measurements. Of course there is also the potential benefit of using the SDE to effect enantiopurification of scalemic material in the production process. Accordingly, the implications of the SDE with respect to the production, storage, and administration of chiral drugs should be carefully considered.

### 5.3 Emergence of prebiotic homochirality

**Scheme 6:**
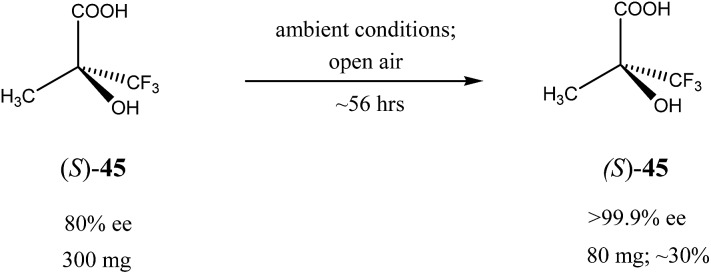
SDE *via* sublimation under ambient conditions in the open air.

One of the most intriguing implications of the SDE phenomenon is its relevance to the emergence of prebiotic homochirality. The origin of homochirality and its role in the development of life on Earth are among the most fundamental, enigmatic, and yet so far, unanswered questions in science. Over the years, many exciting and interesting proposals have been put forward to address the issue of prebiotic homochirality.^93^,^94^ Of these, the generation of chirality *via* autocatalysis^95^ or equilibrating reactions^94^ are of particular scientific excellence. However, all the mechanisms proposed so far require highly tuned, externally controlled conditions. Furthermore, chirogenesis is a process of decreasing entropy,^96^ which, along with the highly controlled conditions, renders these routes of quite low probability by natural occurrence. Moreover, a valid proposal for the origin of homochirality should be applicable not only under the credible conditions of the prebiotic Earth, but also to account for the noticeable ee found in meteorites and interstellar ices of α-methyl α-amino acids predominant in the *S* enantiomer.^97^ In this regard, a combination of the electroweak parity violation {with theory predicting,^98^ for instance, a minute (∼10^–9^) preference for (*S*)-alanine} in combination with an SDE process has particular appeal as the SDE is entropically a neutral process since the decreased entropy of the enantioenriched fraction is balanced by the increased entropy of the enantiodepleted portion. Accordingly, in many cases the SDE occurs spontaneously without the requirement of any type of specially controlled conditions. One notable example^21^ is portrayed in Scheme 6 for the SDE *via* sublimation of α-trifluoromethyl lactic acid (**45**).

Due to its very high volatility, induced by the trifluoromethyl group, compound **45** readily sublimes under ambient conditions in the open air, a perfect model to demonstrate the principle of spontaneous SDE generating enantioenriched and -depleted samples. Thus, by simply being left exposed in the open air, a sample of **1** of 80% ee produced enantiopure residue with a 30% yield of the excess enantiomer. It was shown, in accordance with conglomerate behavior, that racemic crystals of lactic acid **1** sublime considerably faster in comparison to the enantiopure crystals, thereby accounting for the observed extraordinary outcome.

**Scheme 7:**
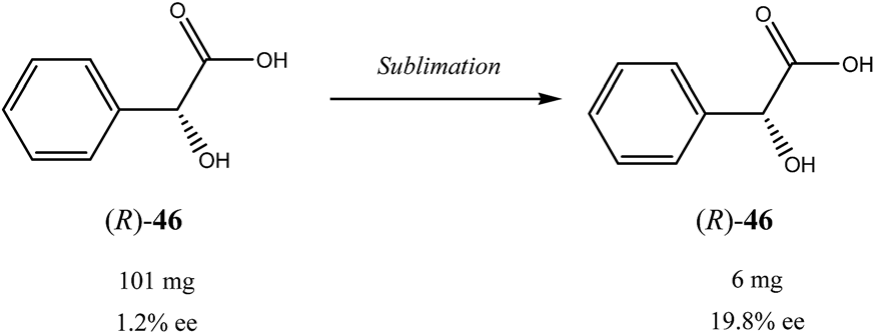
The SDE *via* sublimation of mandelic acid (**46**) of low initial ee.

Another quite remarkable feature of SDE *via* sublimation is illustrated Scheme 7. In this case it was established that a single sublimation step was sufficient to sublime virtually all of the excess enantiomer starting from a sample of mandelic acid (**46**) of very low (1.2% ee) enantiopurity.36*b* This example is also particularly relevant to the issue of prebiotic homochirality showcasing the pathway from materials of minute ee to fractions of synthetically, catalytically meaningful enantioenrichment.

One might agree that sublimation would be one of the most anticipated processes in the vacuum of space where the volatility of all organic compounds is an issue. In this regard, we should also mention some numerous examples of SDE *via* sublimation of protected, as well as zwitterionic, amino acids.36*a* While a comprehensive study in this area is clearly needed, all of the reported data clearly underscore^6^,^21^,^36^,^43^,^99^ the SDE as one of the, if not the, most plausible mechanisms for the generation of prebiotic homochirality.

## 6 Summary

In this minireview, we have reported on the well-documented, yet generally not widely known, SDE phenomenon, the spontaneous fractionation of a scalemate into enantioenriched and -depleted fractions when a physicochemical process – any physicochemical process – has been applied to the scalemate. Examples were presented where the SDE phenomenon can potentially be a great hindrance, especially in cases of ignorance, in terms of altering the ee of samples and the consequent erroneous reporting of ee values as well as miscomprehension of the reaction pathway based on erroneous ee values and other aspects of chiral-based studies. Errors both in the sense of Type I and Type II, *i.e.* methods which are purported to give good stereoselectivity but do not as well as methodologies which are discarded (or used wrongly for interpretations) due to stereoselectivities which were evaluated as poor but which in fact are much better than realized, can occur. Thus, under these circumstances the SDE phenomenon constitutes a decided menace. On the other hand, examples were also presented where the SDE phenomenon can be a potential benefit in terms of a means to effect enantiopurification, even to the point of rivaling conventional techniques for enantiopurification, not only for analytical samples, but also on a preparative scale. In terms of practical application, all forms of liquid chromatography, whether it be analytical HPLC, MPLC, gravity-driven column, flash, or SEC, but in particular, MPLC and SEC, provide opportunities of great potential that workers can take advantage of. For GC, given the difficulty of even effecting the SDE deliberately due to the overlapping limits imparted by column overload, the SDE is unlikely to represent an opportunity, and concurrently, unlikely to represent a menace except in the most exceptional of circumstances. Hence SDE *via* GC can be considered benign and of research interest only to a select band of specialists, otherwise it represents an event of just novelty value for workers at large. But the SDE phenomenon as also representing an opportunity is clearly evident.

The potential implications of the SDE phenomenon are of relevance to any area involving chirality – natural products, asymmetric synthesis, *etc.* The overall outlook is most promising and profound, especially for the potential of the SDE phenomenon to effect enantiopurification, but also for workers to heed the warnings regarding the possibility of erroneous results arising due to the SDE phenomenon altering ee's and to take note of the recommendations put forth here and elsewhere regarding the need for SDE tests^65^ and the rigorous reporting and description of applied physicochemical processes. Though advances have been made in SDE predictability, *e.g.* the concept of SDE-phoric groups,^7^ challenges remain and this review has updated the current situation. In addition, new directions in the study of SDE, including halogen bonding-based interactions and novel, unconventional enantiopurification methods such as pseudo-SDE, have also been reported. Nevertheless, it is worth recounting the following precepts:

• The SDE occurs under totally achiral conditions of: (a) precipitation, (b) centrifugation, (c) evaporation, (d) distillation, (e) crystallization, (f) sublimation, and (g) achiral chromatography (*e.g.* gravity-driven column, flash, MPLC, HPLC, SEC, GC, *etc.*).

• The SDE cannot be controlled simply by experimental accuracy and ignorance of the SDE unavoidably leads to mistakes in the recorded and reported stereochemical outcome of enantioselective transformations.

• The magnitude of the SDE can be controlled and used to: (a) minimize mistakes in the recorded experimental values and (b) to develop unconventional and preparatively superior methods for enantiopurification.

• The magnitude of the SDE cannot be predicted but can be expected for compounds possessing SDE-phoric groups or which have a general tendency for strong hydrogen or halogen bonds or dipole–dipole or aromatic π–π interactions.

• An SDE test^65^ and the rigorous reporting and description of applied physicochemical processes should become part of standard experimental practice to prevent the erroneous reporting of the stereochemical outcome of enantioselective reactions and the chirooptical properties of scalemates.

Whilst the SDE phenomenon is in itself an interesting field of study, it, moreover, provides explanations for other areas of chiral-based phenomena such as NLE's in reactions and NLE's in physicochemical properties such as spectroscopy and could potentially have been an accessorial process leading to the existence of prebiotic homochirality. What is most imperative, and which cannot be stressed too much, is that workers need to take on board the recommendations for SDE tests^65^ and heed the warnings that have been made whenever work involves scalemates that are subjected to any physicochemical process.

## Conflicts of interest

The authors declare there are no conflicts of interest.
